# Geographical characteristics and influencing factors of the influenza epidemic in Hubei, China, from 2009 to 2019

**DOI:** 10.1371/journal.pone.0280617

**Published:** 2023-11-27

**Authors:** Mengmeng Yang, Shengsheng Gong, Shuqiong Huang, Xixiang Huo, Wuwei Wang

**Affiliations:** 1 College of Urban and Environmental Sciences, Central China Normal University, Wuhan, China; 2 Hubei Provincial Center for Disease Control and Prevention, Wuhan, China; University 20 Aout 1955 skikda, Algeria, ALGERIA

## Abstract

Influenza is an acute respiratory infectious disease that commonly affects people and has an important impact on public health. Based on influenza incidence data from 103 counties in Hubei Province from 2009 to 2019, this study used time series analysis and geospatial analysis to analyze the spatial and temporal distribution characteristics of the influenza epidemic and its influencing factors. The results reveal significant spatial-temporal clustering of the influenza epidemic in Hubei Province. Influenza mainly occurs in winter and spring of each year (from December to March of the next year), with the highest incidence rate observed in 2019 and an overall upward trend in recent years. There were significant spatial and urban-rural differences in influenza prevalence in Hubei Province, with the eastern region being more seriously affected than the central and western regions, and the urban regions more seriously affected than the rural region. Hubei’s influenza epidemic showed an obvious spatial agglomeration distribution from 2009 to 2019, with the strongest clustering in winter. The hot spot areas of interannual variation in influenza were mainly distributed in eastern and western Hubei, and the cold spot areas were distributed in north-central Hubei. In addition, the cold hot spot areas of influenza epidemics varied from season to season. The seasonal changes in influenza prevalence in Hubei Province are mainly governed by meteorological factors, such as temperature, sunshine, precipitation, humidity, and wind speed. Low temperature, less rain, less sunshine, low wind speed and humid weather will increase the risk of contracting influenza; the interannual changes and spatial differentiation of influenza are mainly influenced by socioeconomic factors, such as road density, number of health technicians per 1,000 population, urbanization rate and population density. The strength of influenza’s influencing factors in Hubei Province exhibits significant spatial variation, but in general, the formation of spatial variation of influenza in Hubei Province is still the result of the joint action of socioeconomic factors and natural meteorological factors. Understanding the temporal and spatial distribution characteristics of influenza in Hubei Province and its influencing factors can provide a reasonable decision-making basis for influenza prevention and control and public health development in Hubei Province and can also effectively improve the scientific understanding of the public with respect to influenza and other respiratory infectious diseases to reduce the influenza incidence, which also has reference significance for the prevention and control of influenza and other respiratory infectious diseases in other countries or regions.

## 1. Introduction

Influenza is an acute respiratory infectious disease caused by influenza virus, which is mainly transmitted by air, droplets and contact. Influenza virus is easy to mutate, the incubation period is short, infectivity is strong, the disease can occur year-round, the population is generally susceptible, so the incidence rate of influenza is high. Since the 20th century, there have been several influenza pandemics in the world, such as the "Spanish Flu" in 1918, the "Asian Flu" in 1957, the "Hong Kong Flu" in 1968, and "Influenza A" in 2009, all of which were important problems threatening global public health and global economy. Currently, influenza is the first infectious disease under global surveillance by the World Health Organization (WHO). Although it is included in infectious disease management around the world, approximately 1 billion people are still affected by influenza each year, of which 3 to 5 million cases are severe, with 290–650,000 deaths [1]. Therefore, complementing and improving influenza-related research can help improve human health.

Meanwhile, influenza epidemics have distinct seasonal and spatiotemporal clustering characteristics, showing diverse spatial distribution patterns. In temperate regions, influenza occurs mostly in winter (December to March in the Northern Hemisphere and May to September in the Southern Hemisphere), while tropical and subtropical regions have two peak periods of influenza incidence in winter and summer. Spatiotemporal analysis of the U.S. study found that HH clusters and high-risk states were mainly located in the Mississippi, and time clusters were mainly concentrated from January to March [2]. Influenza often begins in the southern United States and progresses to the north and west [3]. The study in China showed that regions with higher influenza incidence were concentrated in northwestern and northern areas and coastal areas in southeastern China. The distribution of regions with higher influenza incidence shifted from the west to the east of the country over time [4]. In the United States, there were approximately 45 million illnesses and 810,000 influenza-related hospitalizations in the 2017–2018 influenza season [2], which is much higher than in previous flu seasons. Research shows that the number of influenza cases reported globally in the 2017–2018 influenza season ranks second after the 2009 H1N1 pandemic [5]. Opting for the timeframe of 2009–2019 for the research, as it facilitates an enhanced comprehension of the spatiotemporal dynamics of influenza epidemics in Hubei Province over the long term. This period also provides insight into the influencing factors behind the high incidence of influenza recorded during the 2017–2018 epidemic season.

Numerical studies have shown that influenza prevalence has obvious seasonality, which is consistent with seasonal characteristics of climate factors to some extent. Studies in China have indicated that low winter temperatures are associated with influenza infection. With the decrease in temperature, the incidence of influenza presents a gradually increasing trend [6]. This is because when temperatures drop or other environmental factors increase the cumulative effect, the regulatory mechanisms of the human body cannot meet the requirements, increasing the risk of disease. Other research has shown that both low and high temperatures are risk factors for influenza A, resulting in two seasonal peaks in the winter/spring and summer months for influenza A in most years [7]. Recent animal experiments have shown the low relative humidities produced by indoor heating and cold temperatures as characteristics of winter that favor the spread of influenza virus in temperate regions [8], and transmission was highly efficient at low RH values of 20% or 35% [9]. In tropical and subtropical regions, the opposite is true. An influenza survey conducted in Dakar showed high transmission during the hot and rainy season [10], and monthly influenza notifications from 67 weather stations across Vietnam also indicated that high rather than low absolute humidity in summer is associated with increased influenza activity [11]. In addition to temperature and humidity, other climatic factors are also thought to be drive the prevalence of influenza. For example, solar radiation may play a role, as varying exposure to solar UV radiation is known to affect the inactivation of viruses in the environment [12]. During autumn and winter, reduced exposure to sunlight affects immune function by altering endogenous production of specific immunomodulators [13]. Direct skin exposure to UV radiation may favor the production of vitamin D3, a regulator of the human immune system. As a result, winter can cause a decreased vitamin D3 levels, making residents in temperate regions more susceptible to infection [14]. Conclusions regarding the correlation between rainfall and influenza activity are inconsistent. Studies in Spain have found that an increase of 10 mm in weekly rainfall equates to a 17% increase in weekly influenza [15]. Higher rainfall would increase indoor crowding and increase the risk of contact transmission in subtropical monsoon areas [16]. However, a study of influenza-associated hospitalization rates in Suzhou, China, found no link between influenza and rainfall [17]. In addition, particulate matter (PM) is also a risk factor for influenza, as influenza virus is attached to atmospheric particles and inhaled by people, and the health impacts of PM pollutants vary by particle size [18]. The relationship between influenza and air pollution in China has been observed. The contents of five air pollutants were significantly positively correlated with influenza incidence, with a decreasing contribution order of SO2 > CO > NO2 > PM2.5 > PM10 [4]. Thus, climatic factors can influence the spread of influenza by affecting the immunity, behavior, and survival rate of the virus outside the human body. Compared to a large number of studies on the impact of meteorological factors on influenza, there are relatively few socioeconomic studies on influenza. These studies focused on the roles of human mobility, health care conditions, family economic income, and other socioeconomic factors in the global and regional spread of influenza. They suggested that high transmission caused influenza to spread rapidly beyond local spatial constraints. Work commutes have played a major role in the spread of influenza outbreaks in the United States over the past 30 years [19]. Long-haul airline travel, such as international air travel, exerted an important influence on the introduction of influenza [20]. Crowding of populations also makes an important impact on populations susceptible to influenza, and closed clusters of places such as schools, childcare facilities, and air travel are conducive to influenza epidemics [21, 22]. Another analysis found that race, higher poverty, and the percentage of female-headed households were consistently associated with higher rates of influenza hospitalization [23–25]. Hence, socioeconomic factors primarily affect susceptible populations to spread influenza. Once the new influenza virus, suitable weather factors, and susceptible people appear at the same time, an influenza outbreak is easily caused. Although influenza research is substantial, most studies are limited to their respective epidemiology, virology and immunology domains, and the comprehensive study of human geography in the spatial-temporal distribution of influenza and its influencing factors is limited.

Hubei Province is located in the central region of China, within the mid-latitudes and the middle reaches of the Yangtze River at 108°21′42″-116°07′50″E, 29°01′53″-33°06′47″N, covering an area of 18590000km^2^. As a transitional zone between China’s north and south, its distinct geographical location contributes to the complexity of influenza pathogenesis in the region. Although it comprises only 1.94% of China’s total land area, its permanent population accounts for 4.14% of the national population (2022), resulting in a high population density and a correspondingly high influenza incidence rate. In fact, from 2017 to 2019, the number of influenza cases in Hubei Province increased rapidly from 35,767 to 274,611, posing a significant health concern for the population. Despite this, there are limited research achievements on influenza in the province, with existing studies primarily focused on population distribution, seasonal patterns, and pathogen analysis [26–28]. As such, from the perspective of health geography, this article uses a time series seasonal decomposition model and geographic spatial-temporal analysis to comprehensively assess the spatial-temporal distribution characteristics and influential factors of influenza epidemics in Hubei Province from 2009 to 2019. The findings of this study have important implications for understanding and identifying the spatial-temporal distribution patterns of influenza and the impacts of geographic factors in mid-latitudes. Furthermore, they contribute to the establishment of reasonable and scientifically-based measures to improve public health and prevent future influenza pandemics, such as promoting the administration of influenza vaccines among susceptible populations prior to December each year, particularly in areas with higher influenza incidence. This research also holds significant reference value for influenza prevention and control efforts in other countries and regions.

## 2. Data sources and research methods

### 2.1 Data sources

#### 2.1.1 Influenza incidence data

The number of influenza incidences in 103 counties in Hubei Province from 2009 to 2019 was obtained from the Hubei Disease Prevention and Control Information System (http://www.hbcdc.com/), and the incidence rate was calculated based on the number of incidences and the population. Based on the purpose of the study and data availability, provincial-scale influenza data from 2009 to 2019 were used to analyze temporal changes in influenza epidemics in Hubei Province; county-scale influenza data from 2009 to 2019 were used to analyze the spatial differentiation of influenza epidemics in Hubei Province; and county-scale average incidence data from 2017 to 2019 were used to analyze the influencing factors of influenza epidemics in Hubei Province because Hubei has entered a high influenza season every year since 2017.

#### 2.1.2 Influencing factor data

Infectious disease epidemics are the result of a combination of three factors: pathogens, transmission routes and susceptible populations (Table 1). First, with regard to pathogen factors, the survival and reproduction of influenza virus and its transmission are mainly governed by climatic conditions, so annual mean temperature (*x*_1_), annual average relative humidity (*x*_2_), annual precipitation (*x*_3_), annual mean wind speed (*x*_4_), and annual sunshine hours (*x*_5_) were selected for analysis in this study. 2017–2019 meteorological data were obtained from China Meteorological Data Network (http://data.cma.cn/). Second, regarding the factors related to the transmission route of influenza, it is mainly transmitted through contact transmission and is linked to population distribution and mobility. Based on the research objectives and availability of data, this study selected factors such as permanent resident population (*x*_6_), population density (*x*_7_), urbanization rate (*x*_8_), and road density (*x*_9_) for analysis. Finally, with regard to susceptible population, the population is generally susceptible to influenza, especially children and adolescents, and schools are the places where children and adolescents are concentrated and where influenza is highly prevalent. The immunity of the population to influenza is mostly limited by the regional health care, the education level of the population and the economic conditions of the family, so number of beds per 1,000 population (*x*_10_), number of health technicians per 1,000 population (*x*_11_), number of schools (*x*_12_), number of primary school students in school (*x*_13_) and per-capita disposable income of urban residents (*x*_14_) were selected in this study for analysis. In this study, socioeconomic data from 2017 to 2019 were obtained from various *County Statistical Yearbooks of Hubei Province* and *China County Statistical Yearbooks*.

**Table 1:** Index system of influenza influencing factors in Hubei Province.

Target layer	Criterion layer	Indicator layer and unit	Data sources
Pathogens	Climatic conditions	Annual mean temperature (*x*_1_/°C); Annual average relative humidity (*x*_2_/%); Annual precipitation (*x*_3_/mm); Annual mean wind speed (*x*_4_/m/s); Annual sunshine hours (*x*_5_/h);	Meteorological data for each district and county are obtained from China Meteorological Data Network(http://data.cma.cn/). Use the Kriging interpolation method in Arc GIS software to interpolate data from various meteorological stations, and divide the data into different districts and counties in Hubei Province.
Transmission routes	Contact transmission	Permanent resident population (*x*_*6*_/Person); Population density (*x*_*7*_/People per square kilometer); Urbanization rate (*x*_*8*_/%); Road density (*x*_*9*_/Kilometers per square kilometer);	Permanent resident population (*x*_*6*_), population density (*x*_*7*_) and urbanization rate (*x*_*8*_) data for each district and county are obtained from t*he County Statistical Yearbook of Hubei Province and China County Statistical Yearbook 2018*, *2019 and 2020*, which are statistical data. Road data for each district and county are obtained from Resource and Environment Science and Data Center of the Chinese Academy of Sciences (https://www.resdc.cn), which is a vector data. Road density (*x*_*9*_) is calculated by first obtaining the length of four road types: railways, national roads, provincial roads and county roads in a county-level administrative region through the Intersection tool of ArcGIS software, and then dividing the total road length by the land area.
Susceptible populations	The immunity of susceptible populations	Number of beds per 1,000 population (*x*_*10*_/Bed); Number of health technicians per 1,000 population (*x*_*11*_/Person); Number of schools (*x*_*12*_/School); Number of primary school students in school (*x*_*13*_/Person); Per-capita disposable income of urban residents (*x*_*14*_/Yuan).	These data (*x*_*10*_, *x*_*11*_, *x*_*12*_, *x*_*13*_, *x*_*14*_) for each district and county are obtained from *the County Statistical Yearbook of Hubei Province and China County Statistical Year*book *2018*, *2019 and 2020*, which are statistical data.

### 2.2 Research methods

#### 2.2.1 Spatial and temporal influenza characterization methods

*(1) Time series seasonal decomposition model*. Based on the confirmation of the existence of seasonal influenza incidence, a seasonal decomposition model was used to analyze the seasonality and long-term trend of influenza incidence in Hubei Province. Infectious disease time series are generally composed of four elements: long-term trend (*T*), seasonal variation (*S*), cyclical variation (*C*) and random variation (*R*). In general, the most commonly used methods are additive and multiplicative models, which are used if the magnitude, trend and period of seasonal variation in incidence vary over time; otherwise, additive models are used. Referring to existing studies [29] and the characteristics of the influenza incidence time series, this study used a multiplicative model (*X*_*i*_ = *T* × *C* × *S* × *R*) to decompose the monthly influenza incidence rate. The specific steps are as follows:

First, the moving average *MA*_*i*_ of the 12-month influenza incidence rate was calculated as follows.

$${MA}_{i}=(\frac{1}{2}X_{i-6}+X_{i-5}+\ldots +X_{i+5}+\frac{1}{2}X_{i+6})/12$$ (1)

where *X*_*i*_ denotes the influenza incidence rate in month *i*. Cyclic variation and long-term trend series were obtained by moving averages to eliminate seasonal and random variation; hence, *MA*_*i*_ = *T*×*C*.

Second, the seasonal irregularity component *SR*_*i*_, which contains both seasonal and stochastic factors, was calculated as follows.


$$SR_{i}=X_{i}/MA_{i}=T\times C\times S\times R/T\times C=S\times R$$ (2)

Then, *SR*_*i*_ was decomposed into seasonal factor *S* by eliminating the randomness factor *R* with the monthly mean and normalized to obtain the average state and pattern of influenza incidence over time.

Finally, the *MA*_*i*_ serial values were curve-fitted to obtain the long-term trend *T* equation of the influenza incidence rate, aiming to reflect the overall fluctuation pattern of the influenza incidence rate.

*(2) Spatial autocorrelation analysis*. Spatial autocorrelation analysis is mainly used to check whether the attribute values of spatial units are correlated with those of their neighboring units and is an important method to measure the degree of clustering of the attribute values of spatial units, which is divided into global spatial autocorrelation analysis and local spatial autocorrelation analysis [30].

Global spatial autocorrelation is used to analyze the overall characteristics of the spatial distribution of influenza in Hubei Province, which is measured by Moran’s *I*. Moran’s *I* index generally ranges from -1 to 1. If Moran’s I is greater than 0 and less than 1, it indicates the existence of positive spatial correlation, which is an agglomerative distribution. If Moran’s I is more than -1 but less than 0, it indicates the existence of a negative spatial correlation, which is a discrete distribution. If Moran’s I is equal to 0, then there is no spatial correlation. It is calculated as follows:

$$I=\frac{n\sum_{i=1}^{n}\sum_{j=1}^{n}w_{ij}(x_{i}-\underline{x})(x_{j}-\underline{x})}{\sum_{i=1}^{n}\sum_{j=1}^{n}w_{ij}\sum_{i=1}^{n}{\left(x_{i}-\underline{x}\right)}^{2}}$$ (3)

In (3), *n* = 103, indicates 103 counties in Hubei Province; *w*_*ij*_ represents the weight matrix; *x*_*i*_ and *x*_*j*_ indicate the influenza incidence rate in county *i* and county *j*, respectively; and *x* represents an average value. The significance of spatial autocorrelation is judged by the standardized statistic Z. The formula is as follows:

$$Z=\frac{I-E(I)}{\sqrt{VAR(I)}}$$ (4)

Generally, according to the normal distribution test value, when Z is greater than 1.96 or Z is less than -1.96(α = 0.05), there is a significant spatial correlation with respect to the incidence rate of influenza in Hubei Province.

The Getis-Ord Gi* (abbreviated as Gi* below) index was used to measure the local spatial autocorrelation of influenza in Hubei Province, which can be used to identify the spatial distribution of hot and cold point regions. The formula is as follows:

$$G_{i}^{*}=\sum_{j=1}^{n}W_{ij}x_{j}/\sum_{j=1}^{n}x_{j}$$ (5)

In (5), *n*, *x*_*j*_, and *w*_*ij*_ have the same meaning as in Eq 4. When Gi* > 0 indicates the presence of hot spots, Gi* < 0 indicates the presence of cold spots. This analysis process was performed in Arc-GIS software.

#### 2.2.2 Methods for analyzing influencing factors of influenza

*(1) Geographical detector*. A geographic detector (GD) can effectively detect the spatial heterogeneity of geographic phenomena and their drivers [31]. In this paper, single factor detection and interaction detection are used to identify factors influencing the spatial differentiation of influenza incidence in Hubei Province. The calculation formula is as follows:

$$q=1-\frac{\sum_{h=1}^{L}N_{h}\sigma_{h}^{2}}{N\sigma^{2}}$$ (6)

where the value of *q* is the decisive indicator for measuring spatial variation in influenza incidence, the value range of *q* is [0,1], and the larger the value of *q* is, the greater the influence of the factor on spatial variation in influenza incidence; *h* = 1,……, *L* represents the number of categorical subregions of the influencing factor; *N*_*h*_ is subregion *h*, and *N* is the number of spatial units. Here, *N* = 103 indicates 103 counties in Hubei Province; $\sigma_{h}^{2}$ and *σ*^2^ are discrete differences in influenza in subregion *h* and Hubei Province, respectively.

*(2) Geographically weighted regression analysis*. The geographically weighted regression model (GWR) is a spatial regression method based on the idea of local smoothness, which can be used to analyze the spatial variability of the degrees of different influencing factors on the influenza rate. This method takes into account the geospatial attributes of the data in the model and is an extension of the traditional multiple regression model, focusing on the variability of regression coefficients within geographic space [32]. The model is expressed as follows:

$$y_{i}=\beta_{0}(u_{i},v_{i})+\sum_{k=1}^{n}\beta_{k}(u_{i},v_{i})x_{ik}+\varepsilon_{i}$$ (7)

where *y*_*i*_ is the dependent variable of the sample, which is the influenza rate of a district or a county; *x*_*ik*_ is the matrix of influencing factors; (*u*_*i*_, *v*_*i*_) is the spatial location coordinates of sample *i*; *β*_*k*_ (*u*_*i*_,*v*_*i*_) is the regression coefficient of influencing factor *k*; and *ε*_*i*_ is the random error term. In this study, geographic weighted regression analysis of influencing factors of influenza rate was performed with the help of ArcGIS software, the kernel type was set as fixed distance, and the bandwidth method was the minimum AIC method.

## 3. Results

### 3.1 Temporal changes of the influenza epidemic in Hubei Province

#### 3.1.1 Seasonal variation characteristics of influenza

**Fig 1:**
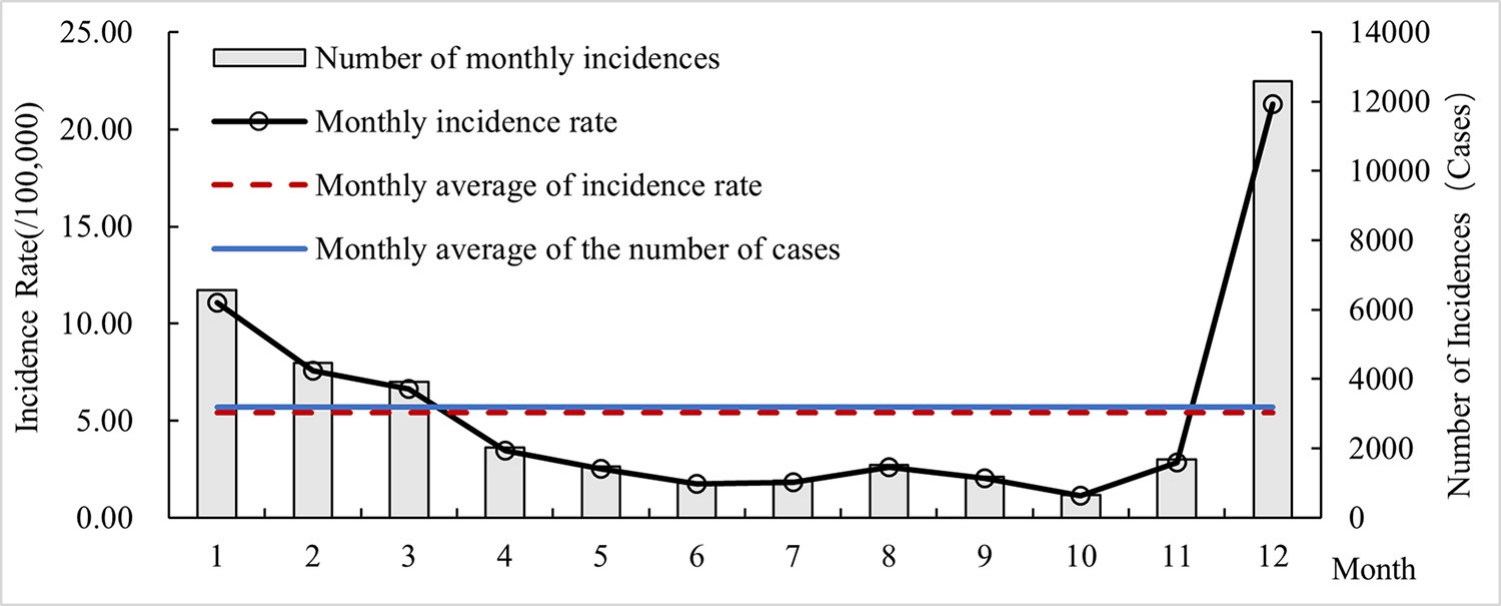
Monthly changes in influenza cases and incidence rates in Hubei from 2009 to 2019.

**Fig 2:**
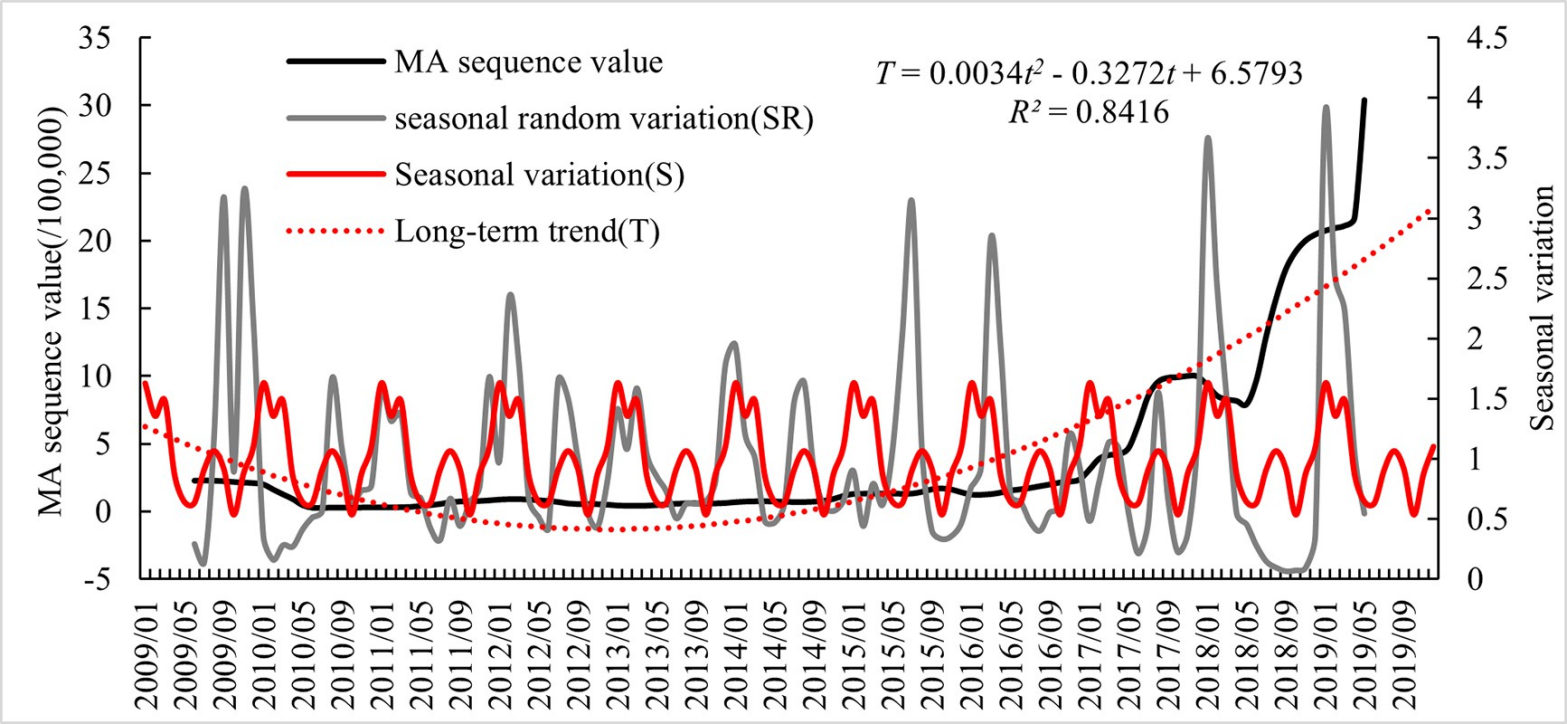
Seasonal index and long-term trend of influenza incidence in Hubei in 2009–2019.

From 2009 to 2019, the average monthly number of influenza cases in Hubei Province was 3187, and the average monthly incidence rate was 5.40/100,000, with a significantly higher number/rate of influenza incidence from December to March (Fig 1). After time series analysis, the seasonal component *S* of the influenza incidence rate in Hubei Province was obtained to form a peak value from December to March, indicating that influenza in Hubei Province is mainly prevalent in winter. According to a previous study, the peak period of influenza in southern China is summer, and the peak period of influenza in northern China is winter and spring [33]. Hubei Province is located in the south but adjacent to the north, and the influenza epidemic display certain transitional characteristics between the north and the south. Therefore, in addition to the peak epidemic period in winter, there is also a small period of high influenza in August in summer (Fig 2).

#### 3.1.2 Interannual variation characteristics of influenza

**Fig 3:**
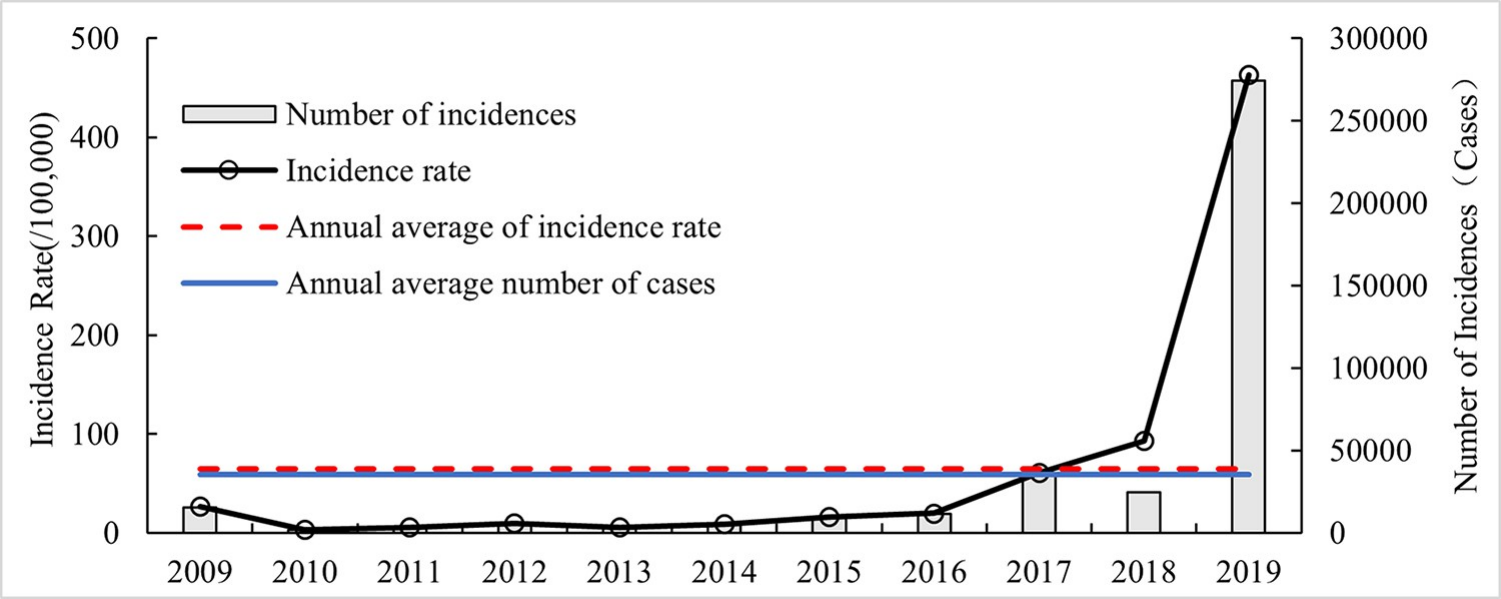
Interannual changes in influenza cases and incidence rate in Hubei from 2009 to 2019.

From 2009 to 2019, the number of influenza cases/incidence rate in Hubei Province decreased and then increased (Fig 3), with the number of cases decreasing from 15,444 in 2009 to 1994 in 2010 and then increasing to 274,611 in 2019, and the incidence rate decreasing from 27.04/100,000 in 2009 to 3.49/1,000 in 2010 and then increasing to 463.31/100,000 in 2019. Both the number of cases and incidence rate in 2019 were the highest in the study period, but there was only a winter peak and no summer peak in that year (Fig 2), which was more concentrated and could be considered a pandemic year for influenza. The reasons for this phenomenon may be related to the absence of two summer peaks in 2018 and 2019 and the occurrence of antigenic mutations of influenza A virus [26].

#### 3.1.3 Long-term trends and characteristics of influenza

After analyzing the time series of the influenza incidence rate in Hubei Province from 2009 to 2019 (Fig 2), the long-term trend equation was obtained as *T* = 0.0034*t*^*2*^–0.3272*t* + 6.5793 (*R^2^* = 0.8416). From this quadratic polynomial equation, it can be determined that the influenza incidence rate in Hubei Province from 2009 to 2019 first declined and then rose, showing an overall upward trend, which has increased in recent years.

### 3.2 Spatial distribution of the influenza epidemic in Hubei Province

#### 3.2.1 Overall distribution characteristics of influenza

**Fig 4:**
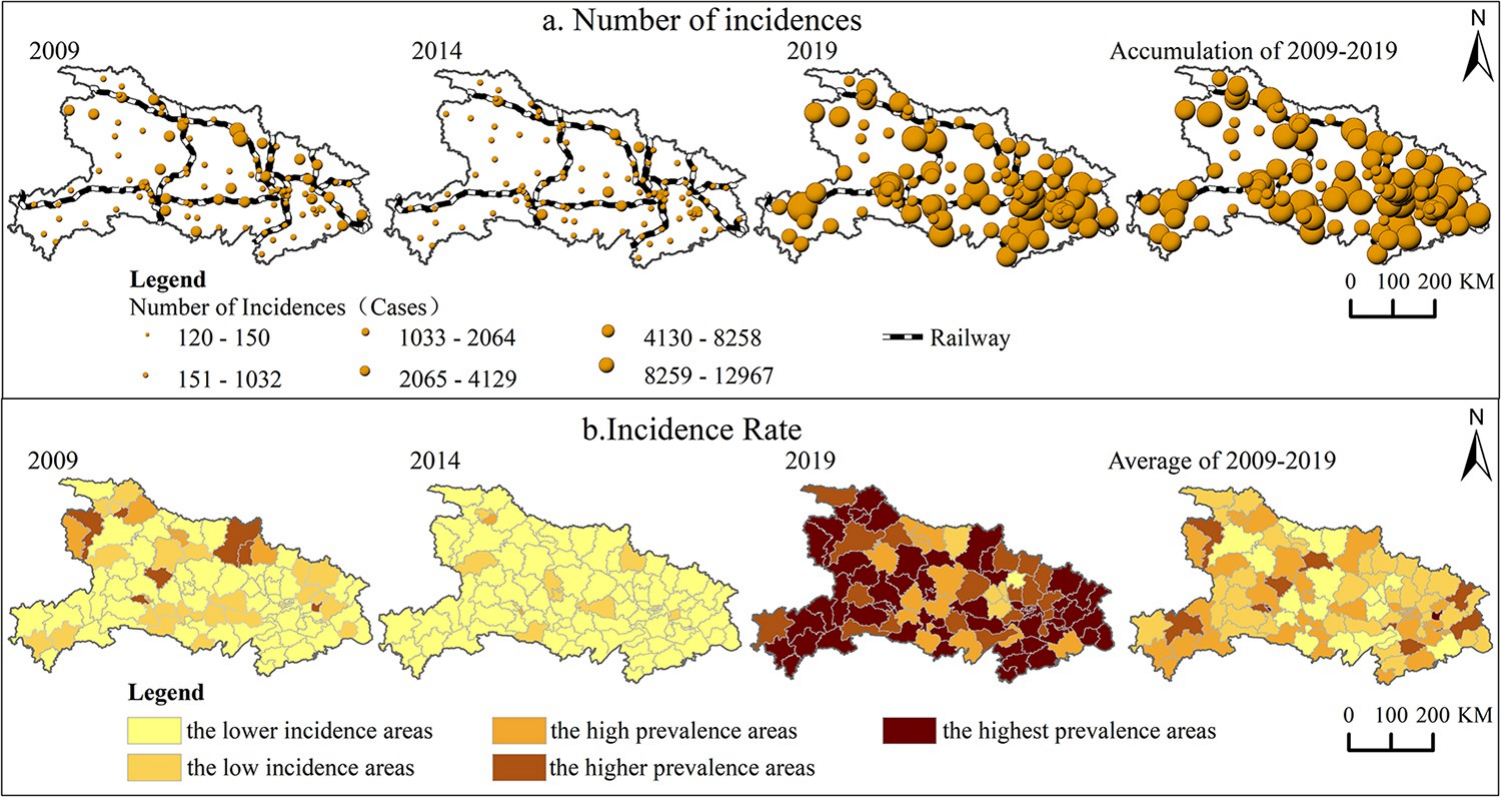
Spatial distribution of influenza incidents in Hubei Province. Note: The basemap came from United States Geological Survey (https://apps.nationalmap.gov/services/), the map boundary has not been changed. Cartographic software: ArcGIS.

*(1) Influenza is widespread*, *but there are significant differences in spatial distribution between regions*. In terms of the number of cases (Fig 4A), influenza in Hubei Province was mainly distributed along the "A-shaped point-axis structure" from 2009 to 2019 [34], with Ezhou, Wuhan, Huanggang, Huangshi, Jingzhou, Yichang, and Shiyan along the route being highly concentrated areas of influenza cases. By region, the proportions of national land area in eastern, middle and western of Hubei Province were 21.73%, 36.19% and 42.36%, respectively, and the corresponding cumulative "concentration" values of influenza cases (the ratio of the proportion of influenza cases to the proportion of national land area) were 2.11, 0.81 and 0.59, respectively. The distribution of influenza cases in the eastern, central and western longitudinal directions is very obvious. By urban and rural areas, the 64 main urban areas and county-level cities are roughly classified as "urban areas", and the remaining districts and counties are classified as "rural areas". The cumulative "concentration" values of influenza cases corresponding to these two types of areas are1.54 and 0.50, respectively, with significantly more cases in urban areas than in rural areas. In summary, the overall distribution of influenza epidemics in Hubei Province is characterized the following: outbreaks in eastern areas are more serious than those in central and western areas, and outbreaks in urban areas are more serious than those in rural areas.

*(2) The scope of the high incidence rate area of influenza gradually expanded*, *and the scope of the low incidence rate area first expanded and then narrowed*. In terms of incidence rate (Fig 4B), the county influenza incidence rate in Hubei Province from 2009 to 2019 was divided into 5 levels according to the natural breakpoint method, and the 5 levels of areas were named with reference to the average annual influenza incidence rate in Hubei Province (64.81/100,000): lower incidence areas (<22.60/100,000), low incidence areas (22.60/100,000–64.81/100,000), high prevalence areas (64.81/100,000–129.62/100,000), higher prevalence areas (129.62/100,000–259.24/100,000), and highest prevalence areas (>259.24/100,000). Among them, in 2009, the "the higher prevalence areas " were scattered in Maohuai, Zhushan, Zengdu, Suixian, Doujun, Yuan’an, Huangzhou and other counties, with the number of counties accounting for only 6.8%, while in 2019, all the provinces were covered with " the highest prevalence areas", with the number of counties accounting for 63.11%, mainly located in Huanggang, Xianning, Wuhan, Shiyan, Enshi, Yichang and other cities in eastern and western Hubei Province. From 2009 to 2019, the number of counties in "the lower incidence areas" accounted for 63.11%, 87.38% and 0.97% respectively, with a trend of spatial distribution expanding first and then narrowing. The spatial distribution of the influenza epidemic in Hubei Province in recent years display a clear trend of spreading, which is consistent with the previous conclusion of temporal changes.

#### 3.2.2 Spatial clustering characteristics of influenza

**Fig 5:**
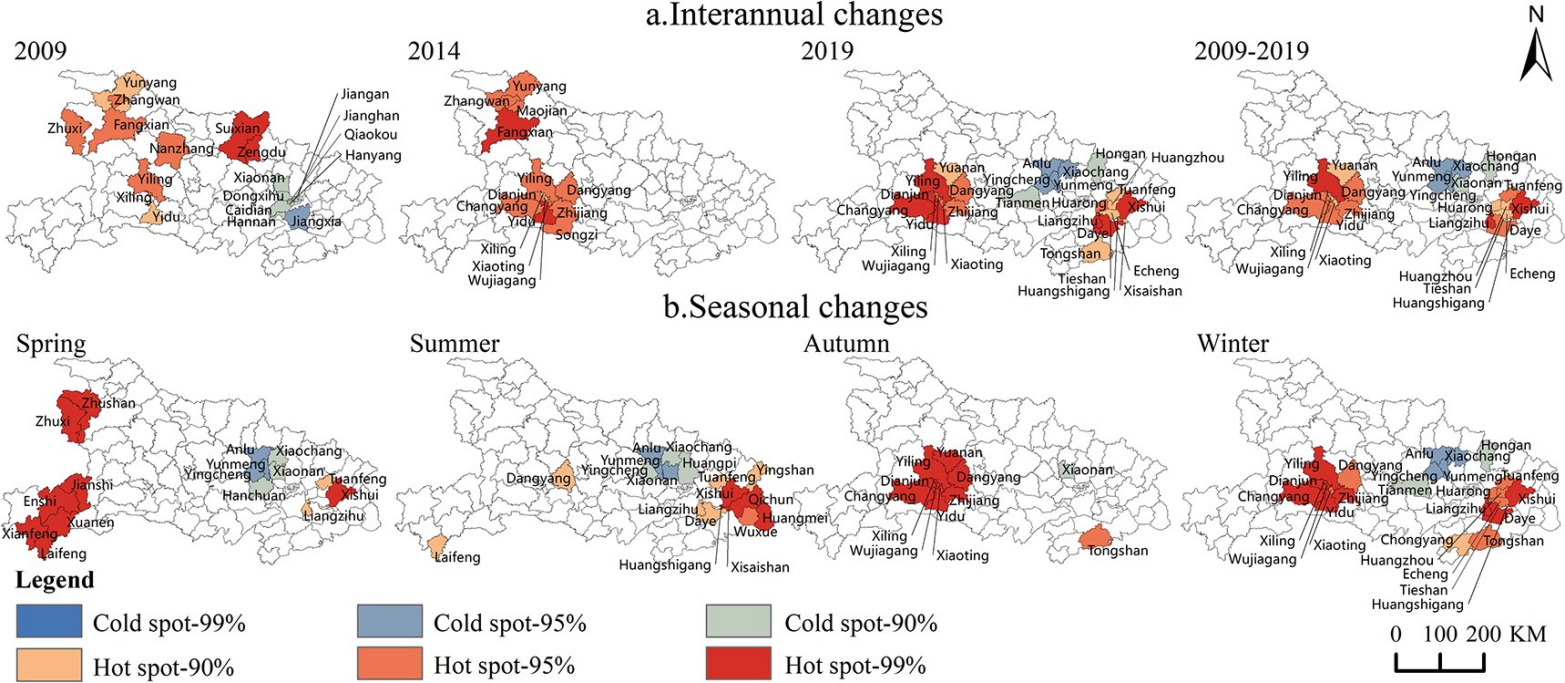
Interannual (a) and seasonal changes (b) of influenza hot spots in Hubei Province. Note: The basemap came from United States Geological Survey (https://apps.nationalmap.gov/services/), the map boundary has not been changed. Cartographic software: ArcGIS.

*(1) Influenza showed an overall clustered distribution*, *with the strongest clustering in winter*. The global Moran’s *I* index of the influenza incidence rate for 2009–2019 was calculated by using ArcGIS software to explore the interannual and seasonal spatial evolution of the influenza epidemic in Hubei Province. In terms of the interannual distribution, Moran’s *I* test statistics were higher than the critical *Z* value (1.96) at the confidence level of 0.05 during the study period, except for 2014–2015, which indicated that the influenza epidemic in Hubei Province had obvious and positive spatial autocorrelation characteristics during the study period except 2014–2015. This means that the spatial distribution showed obvious agglomeration characteristics. The strongest clustering was observed in 2013 (Moran’s *I*: 0.32, *P* < 0.001). In terms of seasonal distribution, Moran’s *I* test statistics were greater than the critical *Z* value (1.96) at the confidence level of 0.05 in 2017.01, 2017.07–10, 2017.12, 2018.03–04, 2019.03–06, and 2019.09–12 during 2017–2019, indicating obvious and positive spatial autocorrelation characteristics of influenza epidemic during these months. The strongest clustering occurred in 2019.12 (Moran’s *I*:0.48, *P* < 0.001). In short, the influenza epidemic in Hubei Province showed a clustered distribution, with the strongest degree of clustering in winter.
*(2) Influenza hot spots were located in eastern and western Hubei Province*, *and cold spots were located in the north-central part of Hubei Province*. In terms of interannual changes (Fig 5), hot spots migrated to the southeast and cold spot areas moved slightly north. In 2009, hot spots were distributed in western Hubei Province, with 9 counties and districts, including Suixian, Zengdu, Zhuxi, Zhangwan, Fangxian, Yunyang, Nanzhang, Yiling, Yidu, and cold spots were distributed in the suburbs of Wuhan. In 2014, hot spots were distributed in 14 counties and districts in the northwest and southwest of Hubei Province, there are four counties and districts such as Fangxian, Zhangwan, Yunyang, Maojian in the northwest of Hubei, and ten counties and districts such as Yidu, Yiling, Dangyang, Zhijiang, Songzi, Dianjun, Changyang, Xiling, Xiaoting, Wujiagang in the southwest of Hubei, and there were no cold spots. In 2019, hot spots in southwest Hubei Province still existed, with newly added hot spots such as Xishui, Daye, Liangzihu, Huangzhou, Tuanfeng, Tieshan, Echeng, Huangshigang, Xisaishan, Tongshan in southeast Hubei Province, and cold spots were distributed in north-central Hubei Province, with 6 counties and districts, including Anlu, Xiaochang, Yingcheng, Yunmeng, Tianmen, Hongan. Throughout the 2009–2019 study period, there were two influenza hot spots in western and eastern Hubei Province, and cold spots were mainly distributed in north-central of Hubei Province. In terms of seasonal changes, the distribution of cold and hot spots of seasonal influenza epidemics in Hubei Province in 2017–2019 was similar to the interannual distribution, with hot spots in spring and autumn mainly distributed in western Hubei Province, hot spots in summer mainly distributed in southeast Hubei Province, and two hot spots in winter, southwest and southeast Hubei Province; cold spots were stable in north-central Hubei Province. Influenza epidemics are often caused by a combination of factors that are related to socioeconomic factors such as population movement and concentration, and may also be related to the large temperature difference between winter and spring in hilly areas of western Hubei Province.

### 3.3 Influencing factors of the influenza epidemic in Hubei Province

#### 3.3.1 Factors influencing temporal changes in influenza

*(1) Factors influencing seasonal changes in influenza*. Meteorological factors can affect the seasonal spread of influenza by influencing the survival rate of the virus outside the human body, transmission routes, immunity of the population, and behavioral habits [35]. First, the monthly influenza incidence rates in Hubei Province from 2009 to 2019 and the monthly mean temperature, precipitation, relative humidity, wind speed and sunshine hours during the same period were correlated. The results showed that the overall influenza incidence rate was negatively correlated with the monthly average temperature (*r* = -0.19) at a confidence level of 0.05, a relatively more significant value, indicating that influenza occurred more frequently in cold weather. Second, the influenza incidence rates of each district and county in Hubei Province from 2017 to 2019 and the monthly mean temperature, precipitation, relative humidity, wind speed, and sunshine hours of each district and county during the same period were analyzed for correlations. The results showed (Table 2) that the influenza incidence rate was correlated with the monthly mean temperature (*r* = -0.30), precipitation (*r* = -0.11), sunshine hours (*r* = -0.24), and wind speed (*r* = -0.07), all of which showed significant negative correlations at the confidence level of 0.01 and significant positive correlations with relative humidity (*r* = 0.08) at the confidence level of 0.01, indicating that influenza is more likely to occur in conditions of low temperature, less rain, less sunshine, low wind speed and humid weather. This is because low temperatures can prolong the survival time of influenza viruses in the environment [36], especially during winter, when cold weather drives people to increase their indoor activities, and lack of air circulation increases the probability of illness, as does cloudy weather; conversely, long daylight hours and high UV intensity can effectively destroy and kill airborne viruses [37]. Influenza viruses are more likely to combine with moisture in the air under high humidity conditions, and the increase in volume makes the rate of decline faster. Combined with poor air mobility under low wind speed conditions, the virus can remain suspended in the air for longer periods of time, thus increasing the likelihood of illness. In addition, the influenza incidence rate is positively correlated with mean temperature in March, June, November, and December, indicating that sudden changes in temperature during seasonal transitions are more likely to trigger influenza prevalence, while seasonal transitions and alternating heat and cold affect the regulation of the body’s thermal function to a certain extent, leading to the occurrence and spread of influenza, especially among the elderly and frail. The influenza incidence rate was significantly negatively correlated with precipitation in March, April, and July and significantly positively correlated with precipitation in June and August, indicating that the relationship between influenza and precipitation is more complex.

**Table 2:** Spearman correlation analysis of influenza incidence in Hubei from 2017 to 2019.

Time	Temperature (*x*_1_)	Humidity (*x*_2_)	Precipitation (*x*_*3*_)	Wind speed (*x*_4_)	Sunshine (*x*_5_)
**January**	-0.51**	0.35**	0.04	-0.13*	-0.41**
**February**	-0.63**	0.32**	-0.08	-0.19**	-0.31**
**March**	0.25**	-0.02	-0.21**	-0.08	0.32**
**April**	-0.10	0.30**	-0.19**	-0.03	-0.41**
**May**	-0.35**	-0.11	0.06	-0.24**	-0.22**
**June**	0.17**	-0.08	0.19**	-0.002	0.07
**July**	-0.15*	0.06	-0.23**	-0.03	-0.12*
**August**	-0.27**	0.34**	0.33**	-0.24**	-0.32**
**September**	-0.12*	0.19**	0.11	-0.14*	-0.12
**October**	-0.02	0.12*	0.11	-0.09	-0.16**
**November**	0.20**	-0.18**	-0.11	-0.11	-0.17**
**December**	0.46**	0.16**	-0.10	-0.17**	0.13*
**Monthly Total**	-0.30**	-0.11**	0.08**	-0.24**	-0.07**

Note: ** indicates that the significance level is 0.01

* indicates that the significance level is 0.05.

*(2) Factors influencing interannual changes in influenza*. Correlation analysis of the annual average influenza incidence rate of each district and county in Hubei Province from 2017 to 2019 with the annual average of 14 influencing factors showed that the influenza incidence rate was significantly and positively correlated with road density (*r* = 0.26), urbanization rate (*r* = 0.26), number of beds per 1,000 population (*r* = 0.23), number of health technicians per 1,000 population (*r* = 0.23) and annual average relative humidity (*r* = 0.20) at the confidence level of 0.05, and number of primary school students in school (*r* = -0.21), annual mean wind speed (*r* = -0.26), permanent resident population (*r* = -0.26), and number of schools (*r* = -0.27) showed a negative correlation at the confidence level of 0.05. A further stepwise regression analysis of 14 influencing factors showed that only road density (*r* = 0.64), urbanization rate (*r* = 0.32), annual mean wind speed (*r* = -0.28), and population density (*r* = -0.68) had a significant impact on influenza incidence (*y* = -0.28*x*_*4*_-0.68*x*_*7*_+0.32*x*_*8*_+0.64*x*_*9*_). All these results indicate that natural environmental factors and socio-economic levels jointly influence interannual changes in influenza incidence rates. Specifically, it is closely related to the influencing factors of influenza pathogen transmission, especially road density, urbanization rate and population density. Therefore, the spread of contact caused by the large-scale movement of people in the global integration environment is one of the reasons for the high incidence of influenza.

#### 3.3.2 Factors influencing the spatial differentiation of influenza

**Fig 6:**
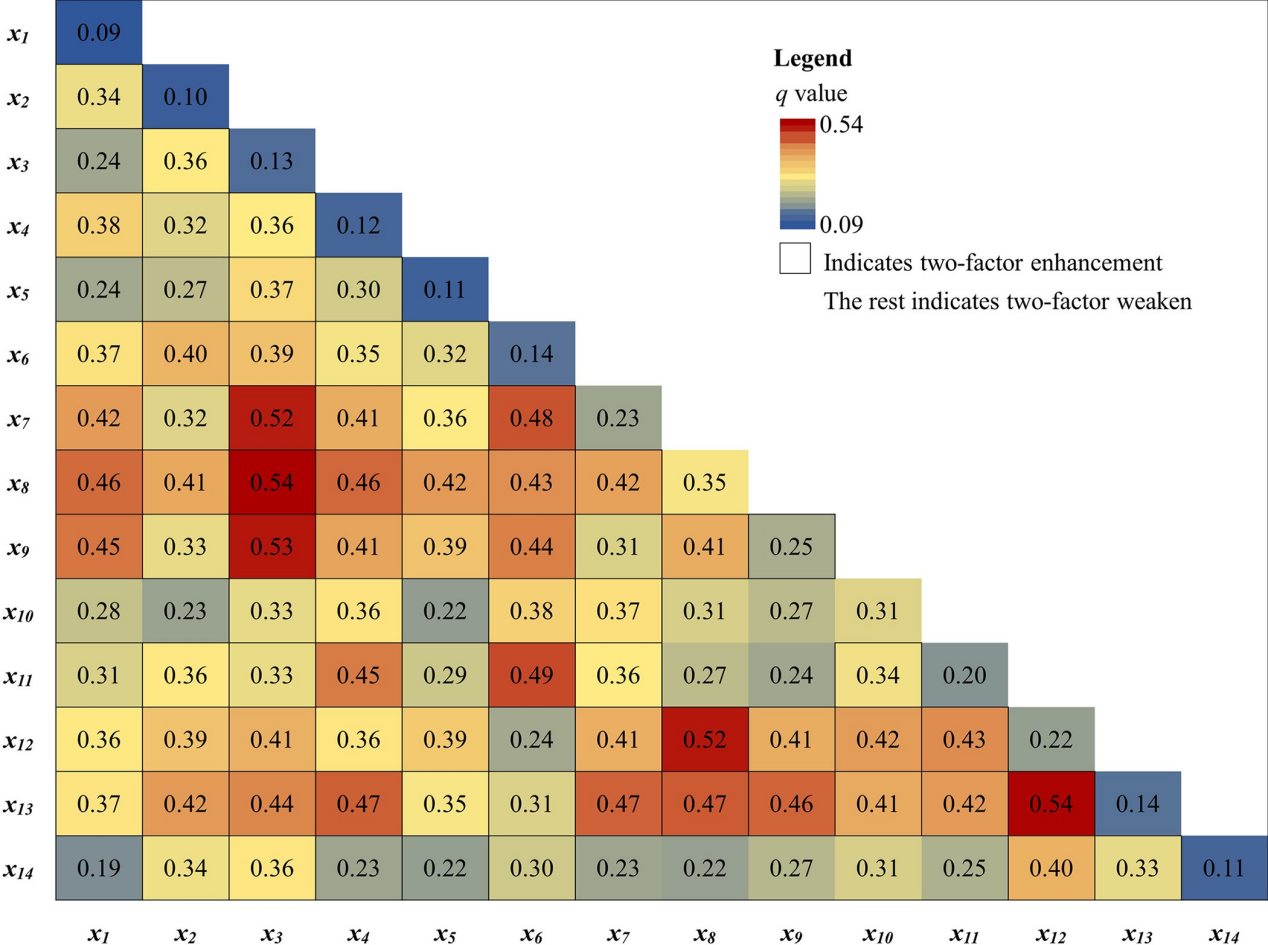
Interaction hotspot maps of influencing factors on influenza incidence in Hubei Province. Note: *x*_*1*_: temperature, *x*_*2*_: humidity, *x*_*3*_: precipitation, *x*_*4*_: wind speed, *x*_*5*_: sunshine, *x*_*6*_: permanent resident population, *x*_*7*_: population density, *x*_*8*_: urbanization rate, *x*_*9*_: road density, *x*_*10*_: number of beds per 1,000 population, *x*_*11*_: number of health technicians per 1,000 population, *x*_*12*_: number of schools, *x*_*13*_: number of primary school students in school, *x*_*14*_: per-capita disposable income of urban residents.

*(1) Effect intensity of influencing factors*. The results of detecting factors influencing spatial changes in influenza incidence rate in Hubei Province by geographical detector showed that, in terms of single-factor detection, the following factors passed the *p* < 0.1 significance test, ranked by *q*-value as urbanization rate (0.35) > number of beds per 1,000 population (0.31) > road density (0.25) > population density (0.23) > number of schools (0.22) > number of health technicians per 1,000 population (0.20) > number of primary school students in school (0.14) > precipitation (0.13) > wind speed (0.12), revealing that the single factor explanatory power of socio-economic factors is stronger. In terms of interaction detection, the interaction between socio-economic factors and natural meteorological factors is significantly stronger, such as interaction between precipitation and urbanization rate (0.54), precipitation and road density (0.53), precipitation and population density (0.52) (Fig 6). Taken together, the formation of spatial changes in the influenza incidence rate in Hubei Province is the result of the combined effect of socioeconomic and natural meteorological factors.

**Fig 7:**
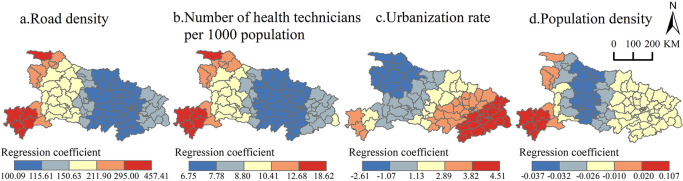
Spatial variability of regression coefficients for influencing factors for influenza incidence in Hubei Province. Note: The basemap came from United States Geological Survey (https://apps.nationalmap.gov/services/), the map boundary has not been changed. Cartographic software: ArcGIS.

*(2) Spatial variation in the degree of influencing factors*. Geographically weighted regression analysis revealed significant spatial variation in the effects of road density, number of health professionals per 1,000 individuals, urbanization rate, and population density on influenza incidence in Hubei Province (Fig 7). The spatial differentiation of the action intensity of road density and population density is similar, which increases in gradient from east to west, with road density having the greatest impact, indicating that the impact of population mobility on influenza epidemic in western Hubei Province is significantly greater than that in central and eastern Hubei Province. The temperature difference is greater in the mountainous and hilly areas of western Hubei Province, which affects thermoregulatory function of the human body, so differences in the natural environment make people in western Hubei Province more susceptible to influenza. While the seasonal population inflow increases in winter and spring when a large number of students and workers return home, the population outflow mainly occurs from eastern Hubei Province, so the effect on western Hubei Province is stronger. Number of health technicians per 1,000 population is positively correlated with the impact of the influenza pandemic, increasing from east to west, indicating that compared to the eastern plains, the health resources in mountainous and hilly areas have a greater impact on the influenza pandemic. Urbanization rate’s role on influenza epidemics decreases from southeast to northwest: the urbanization rate reflects the degree of population concentration, the probability of influenza virus transmission is higher in urban areas with high population concentration [38], urbanization level is higher in eastern Hubei Province, and the intensity of urbanization rate’s role in influenza epidemic is therefore also higher.

## 4. Discussion

Based on the number of influenza incidence cases and incidence rate data in Hubei Province from 2009 to 2019, the spatial and temporal distribution characteristics of influenza epidemics in Hubei Province were analyzed in detail through time series analysis and geographic spatial analysis, and the susceptible seasons and hot spots of influenza epidemics were summarized. In the process of analysis and summary, it is found that influenza epidemics are the result of the combined effect of socioeconomic and natural climate factors. Among them, natural climate factors mainly play a major role in seasonal changes in influenza, while socioeconomic factors exert a dominant force on interannual changes in influenza.

### (1) The temporal variation characteristics of influenza epidemics

Influenza virus has high variability, and large amounts of antigens on the surface of the influenza subtype virus can easily form various subtypes, resulting in a high incidence rate. In addition, there is no cross immunity between subtypes, so seasonal epidemics readily occur. In this study, based on a time series seasonal decomposition model, we analyzed temporal changes of influenza epidemic. First, the seasonal characteristics of influenza epidemics. Influenza epidemics in Hubei Province mainly peak in winter during the year, which is similar to many studies in areas such as Israel [39], Spain [15], and Japan [40], and in the mid-latitude region of Hunan Province, China [41]. In addition to the peak outbreak period in winter, there is also a small high incidence period in August in summer, which shows that the influenza epidemic in Hubei Province exhibits certain north-south transition characteristics, which are similar to the epidemiological characteristics of Shanghai [42]. Secondly, the interannual variation characteristics of influenza epidemics. The cases/rates increased rapidly from 2017 to 2019, similar to the situation in many places around the world during the same period [2–43]. More seriously, approximately 45 million illnesses and 810,000 influenza-related hospitalizations occurred across the United States during the 2017–2018 influenza season. However, the number in 2019 represents the highest in recent years in Hubei Province, which is related to the absence of two short summer periods of high influenza in Hubei Province in 2018 and 2019, and the cyclical influenza A virus epidemic (mini-pandemic occurring on average 2–3 years, with a pandemic every 10–15 years) [26]. Specifically, there was a sharp increase in influenza cases in December 2019 and January 2020, mainly due to the following reasons: First, this month is already a high season for influenza outbreaks. Secondly, since Hubei Province is the first place where COVID-19 has broken out, the influenza epidemic in these two months may overlap slightly with COVID-19. Finally, since the initial symptoms of COVID-19 and influenza are similar, there is a certain probability of misdiagnosis at the initial stage of COVID-19 outbreak. Therefore, influenza prevention and control efforts should focus on strengthening influenza vaccination prior to the winter influenza season, as well as daily cleaning and disinfection of key public places such as schools and markets.

### (2) The spatial clustering characteristics of influenza epidemics

Spatial-temporal analysis is usually used to identify high-risk areas for many diseases. For example, space-time scan statistics were used to detect the high-risk areas of hand, foot and mouth disease [44]. Spatial autocorrelation was used to identify the spatial clustering characteristic of indigenous dengue cases [45]. Spatial-temporal analysis was also used to distinguish hot spots and cold spots of influenza [46]. In this study, the clustering of influenza incidence cases in Hubei Province was mainly concentrated in the eastern and urban areas. Spatial autocorrelation analysis revealed that hot spots had a tendency to migrate from west to east, which is strongly similar to the epidemic of hand, foot and mouth disease in Hubei Province [47], and is highly consistent with the population migration trend in Hubei Province in recent years [48]. Therefore, the priority areas for influenza prevention and control in Hubei Province should be placed along the "A-shaped point-axis structure" cities with high urbanization levels, and the monitoring of migrant populations should be strengthened.

### (3) The influencing factors of influenza epidemics

First, the influencing factors of influenza seasonal prevalence. Transmission of influenza has obvious seasonal characteristics, especially a higher incidence rate in winter and spring, which is consistent with many respiratory diseases [49, 50]. Hubei Province is located in a subtropical monsoon climate region, and most parts experience cold winters, with an average temperature between 2°C and 4°C, hot summers, variable spring temperatures, and rapid fall in autumn temperatures. Studies have shown that higher influenza incidence in temperate regions usually occurs in winter and spring, while in subtropical regions, the seasonal distribution pattern of influenza seems to be somewhat complex. In this study, the low-temperature weather in Hubei Province was found to be favorable for influenza epidemics, which is similar to the United States, Spain and Beijing [2, 15, 51]. It is important to pay attention to influenza viruses during low temperature weather in winter. However, this study also found that the influenza incidence rate was positively correlated with mean temperatures in March, June, November and December. Sudden changes in temperature during the seasonal transition are more likely to trigger influenza epidemics. That is, seasonal transition and alternating cold and heat affect the regulation of the human body’s homeostatic functions to a certain extent. Therefore, extreme weather is also an important reason for the spread of influenza.

Secondly, regarding the influencing factors of influenza spatial differentiation. According to epidemiological principles, the transmission of infectious diseases can be divided into ecological processes and social processes. Spatial proximity allows diseases to spread within cities, and improved intercity transportation systems are conducive to the spread of the epidemic among cities. Therefore, the socioeconomic development of cities is closely related to the process of transmission of infectious diseases. In this study, the influenza incidence rate in Hubei Province was found to be positively correlated with the urbanization rate and road density, which are consistent with factors influencing COVID-19 prevalence [52]. The number of beds per 1,000 population and the number of health technicians per 1,000 population are positively correlated with influenza incidence, which is consistent with factors influencing the prevalence of hand, foot, and mouth disease [47]. All suggest that areas with better economic conditions have higher levels of available health care, higher rates of active medical visits by residents, and higher rates of influenza diagnosis, which may lead to better economic areas having higher influenza incidence rates. This finding was confirmed by other studies [40–53]. Compared to previous influenza studies or known hypotheses, there are also inconsistencies in this study. The influenza incidence rate in Hubei Province is negatively correlated with population density. H1N1 was sustained longer in areas with higher population densities and mobility during the 2009–10 influenza pandemic [54, 55]. Studies in the United States have revealed a positive relationship between mortality rates and population densities [56]. Crowding also contributed to higher mortality than less-crowded areas in Nigeria, indicating that high population density leads to conditions where multiple transmission routes simultaneously operate at a high intensity [57]. However, with the rapid development of the world’s socioeconomic conditions and population density, the establishment of large cities, and the acceleration of the process of world integration, the relationship between population density and the spread of influenza has also become controversial. In Hubei Province, the reason for the negative correlation may be that the deterrent effects of influenza transmission in high-density population areas, such as economic conditions, medical level, and educational level, outweigh the promoting effects of population density on influenza transmission. For example, although the population density in urban areas of Wuhan (Jiangan, Jianghan, Qiaokou, and Wuchang districts) is the largest, the influenza incidence rate is not correspondingly. In addition, the population base and political area of individual areas also affect the relationship between the influenza incidence rate and population density, such as Enshi City, which has a small population base, large political area and relatively low population density, but an influenza incidence rate as high as 156.96 per 100,000, thus representing one of the few areas in the province with the exceptionally highest prevalence. Therefore, it can be seen that the positive effect of socioeconomic factors, especially population density, on infectious disease epidemics also has a threshold space, and in modern society, higher population density is not more likely to lead to influenza epidemics. Future in-depth research is needed to quantify the threshold level of population density in areas with high influenza incidence rates.

## 5. Conclusions

In this paper, based on the data of influenza incidence from the Hubei Province CDC, the spatial-temporal distribution characteristics and influencing factors of influenza in Hubei Province during 2009–2019 were studied by using time series seasonal decomposition analysis, Arc GIS spatial analysis, spatial autocorrelation analysis, geographical detector, and geographically weighted regression analysis. The results have the following implications:

1. Influenza has a high incidence in winter and spring during the year, and of course, there is also a small high incidence period in summer. The overall trend of interannual changes is increasing, reminding us of the need to strengthen attention to influenza viruses. Influenza is prevalent but exhibits significant spatial differences. Influenza is more serious in the eastern region than the central and western regions, while urban areas experience more severe influenza than rural areas. Epidemic hotspots are distributed in the eastern and western regions, and cold spots are located in the central and northern regions. In general, the influenza epidemic in Hubei Province displays significant spatiotemporal clustering.
2. The influenza epidemic in Hubei Province is the result of the combined effect of natural meteorological factors and socioeconomic factors. The seasonal variation in influenza epidemics is mainly influenced by meteorological factors: low temperature, low sunshine, low rainfall, high humidity and low wind speed are favorable to influenza epidemics. The interannual variation in the influenza epidemic is mainly influenced by socioeconomic factors, and the spatial variation in influenza incidence is the result of the interaction between natural meteorological and socioeconomic factors. Factors such as road density, number of health technicians per 1,000 population, urbanization rate, and population density exhibit more significant spatial variation in the influenza epidemic.

## Supporting information

S1 File (DOCX)
