# Supplementary material for: Geographical characteristics and influencing factors of the influenza epidemic in Hubei, China, from 2009 to 2019
Source: PLoS One. 2023 Nov 27;18(11):e0280617. doi: 10.1371/journal.pone.0280617 (PMC10681244; doi:10.1371/journal.pone.0280617)
Supplement: S1 File — (DOCX) [file pone.0280617.s001.docx]

**Geographical characteristics and influencing factors of the influenza epidemic in Hubei, China, from 2009 to 2019**

Mengmeng Yang , Shengsheng Gong , Shuqiong Huang , Xixiang Huo , Wuwei Wang

Supplementary Material

1. **Influenza incidence data in Hubei Province**

In this work, there are 103 districts and counties in Hubei Province. Annual incidence rate at the provincial level in Hubei Province from 2009 to 2019 is shown in Table 1, and the annual incidence rate of 103 districts and counties in Hubei Province in 2009, 2014, and 2019 is shown in Table 2. Based on these data, the spatial and temporal distribution characteristics of influenza epidemic in Hubei Province were obtained through time series analysis and geographic spatial analysis, as shown in Figure 1-5.

**2. Analysis of influential factors of influenza epidemic in Hubei Province**

The data of 14 relevant influencing factors from 103 districts and counties in Hubei Province are shown in Table 3, which is the average value of each influencing factor from 2017 to 2019. Based on data on influenza incidence and influencing factors, the factors influencing seasonal changes in influenza prevalence are analyzed by correlation analysis, that is, the correlation analysis of influenza incidence rate and meteorological factors over the same period. See Table 4 for analysis results. The intensity of interannual changes affecting influenza epidemics was analyzed using geographic detectors. See Figure 6 for analysis results. Geographic weighted regression analysis was used to analyze spatial distribution differences in interannual changes affecting influenza epidemics. See Figure 7 for analysis results.

Table 1: Annual incidence rate at the provincial level in Hubei Province in Hubei Province from 2009 to 2019

| **Time** | **Number of Incidences** |
| --- | --- |
| **2009** | 15444 |
| **2010** | 1994 |
| **2011** | 3113 |
| **2012** | 5474 |
| **2013** | 3469 |
| **2014** | 5065 |
| **2015** | 9201 |
| **2016** | 11610 |
| **2017** | 35767 |
| **2018** | 24988 |
| **2019** | 274611 |

Table 2: The annual incidence rate of 103 districts and counties in Hubei Province in 2009, 2014, and 2019

| **Area** | **Number of incidences in 2009** | **Number of incidences in 2014** | **Number of incidences in 2019** |
| --- | --- | --- | --- |
| Jiangan | 77 | 265 | 8075 |
| Jianghan | 75 | 106 | 3543 |
| Qiaokou | 76 | 74 | 2985 |
| Hanyang | 117 | 57 | 4553 |
| Wuchang | 152 | 82 | 11772 |
| Qingshan | 13 | 22 | 3978 |
| Hongshan | 99 | 21 | 12967 |
| Dongxihu | 23 | 45 | 3737 |
| Hannan | 1 | 23 | 4020 |
| Caidian | 0 | 25 | 927 |
| Jiangxia | 16 | 39 | 8492 |
| Huangpi | 49 | 100 | 2297 |
| Xinzhou | 205 | 46 | 1313 |
| Huangshigang | 66 | 151 | 3173 |
| Xisaishan | 22 | 11 | 1403 |
| Xialu | 23 | 5 | 2574 |
| Tieshan | 7 | 0 | 120 |
| Yangxin | 95 | 21 | 683 |
| Daye | 29 | 16 | 5702 |
| Maojian | 408 | 380 | 2494 |
| Zhangwan | 148 | 87 | 1803 |
| Yunyang | 146 | 21 | 2070 |
| Yunxi | 49 | 58 | 803 |
| Zhushan | 545 | 6 | 3173 |
| Zhuxi | 263 | 49 | 2798 |
| Fangxian | 12 | 9 | 778 |
| Danjiangkou | 503 | 28 | 2573 |
| Xiling | 237 | 120 | 10974 |
| Wujiagang | 129 | 40 | 5771 |
| Dianjun | 108 | 17 | 1171 |
| Xiaoting | 0 | 77 | 635 |
| Yiling | 20 | 21 | 3490 |
| Yuanan | 414 | 1 | 1130 |
| Xingshan | 18 | 1 | 866 |
| Zigui | 7 | 26 | 792 |
| Changyang | 40 | 0 | 1463 |
| Wufeng | 6 | 0 | 383 |
| Yidu | 143 | 1 | 888 |
| Dangyang | 8 | 1 | 535 |
| Zhijiang | 176 | 2 | 507 |
| Xiangcheng | 314 | 55 | 404 |
| Fancheng | 377 | 39 | 926 |
| Xiangzhou | 74 | 12 | 934 |
| Nanzhang | 328 | 13 | 4831 |
| Gucheng | 31 | 2 | 1021 |
| Baokang | 21 | 0 | 249 |
| Laohekou | 50 | 1 | 517 |
| Zaoyang | 6 | 1 | 338 |
| Yicheng | 129 | 18 | 5955 |
| Liangzihu | 17 | 26 | 281 |
| Huarong | 2 | 64 | 640 |
| Echeng | 94 | 126 | 4773 |
| Dongbao | 45 | 89 | 2246 |
| Duodao | 20 | 66 | 1542 |
| Shayang | 5 | 1 | 421 |
| Jingshan | 4 | 34 | 2425 |
| Zhongxiang | 4 | 27 | 630 |
| Xiaonan | 190 | 53 | 1722 |
| Xiaochang | 34 | 14 | 122 |
| Dawu | 12 | 7 | 1429 |
| Yunmeng | 4 | 4 | 227 |
| Yingcheng | 2 | 42 | 188 |
| Anlu | 4 | 0 | 1681 |
| Hanchuan | 120 | 23 | 306 |
| Shashi | 131 | 121 | 5122 |
| Jingzhou | 130 | 34 | 3188 |
| Gongan | 122 | 26 | 1013 |
| Jianli | 18 | 28 | 804 |
| Jiangling | 0 | 2 | 2648 |
| Shishou | 263 | 29 | 4468 |
| Honghu | 1 | 16 | 1233 |
| Songzi | 232 | 179 | 3372 |
| Huangzhou | 787 | 18 | 7867 |
| Tuanfeng | 107 | 7 | 2015 |
| Hongan | 207 | 27 | 1479 |
| Luotian | 63 | 23 | 4933 |
| Yingshan | 72 | 7 | 1444 |
| Xishui | 591 | 10 | 3581 |
| Qichun | 84 | 7 | 8986 |
| Huangmei | 177 | 123 | 3197 |
| Macheng | 286 | 69 | 2351 |
| Wuxue | 382 | 43 | 2227 |
| Xianan | 92 | 25 | 9676 |
| Jiayu | 0 | 5 | 359 |
| Tongcheng | 0 | 37 | 2542 |
| Chongyang | 0 | 0 | 2248 |
| Tongshan | 53 | 2 | 2364 |
| Chibi | 1 | 2 | 2877 |
| Zengdu | 1129 | 88 | 1978 |
| Suixian | 1769 | 183 | 2226 |
| Guagshui | 705 | 313 | 1356 |
| Enshi | 93 | 8 | 9114 |
| Lichuan | 18 | 36 | 1465 |
| Jianshi | 40 | 1 | 2316 |
| Badong | 3 | 3 | 1783 |
| Xuanen | 78 | 15 | 1969 |
| Xianfeng | 96 | 15 | 1685 |
| Laifeng | 6 | 9 | 1651 |
| Hefeng | 23 | 0 | 1349 |
| Xiantao | 415 | 214 | 4045 |
| Qianjiang | 457 | 91 | 1929 |
| Tianmen | 352 | 452 | 5100 |
| Shennongjia | 25 | 26 | 461 |

Table 3: The average data of 14 relevant influencing factors in 103 districts and counties of Hubei Province from 2017 to 2019

| **Area** | Incidence Rate(/100,000) | Annual mean temperature (*x*_1_/℃) | Annual average relative humidity (*x*_2_/%) | Annual precipitation (*x*_3_/mm) | Annual mean wind speed (*x*_4_/m/s) | Annual sunshine hours (*x*_5_/h) | Permanent resident population (*x_6_*/Person) | Population density (*x_7_*/People per square kilometer) | Urbanization rate (*x_8_*/%) | Road density (*x_9_*/Kilometers per square kilometer) | Number of beds per 1,000 population (*x_10_*/Bed) | Number of health technicians per 1,000 population (*x_11_*/Person) | Number of schools (*x_12_*/School) | Number of primary school students in school (*x_13_*/Person) | Per-capita disposable income of urban residents (*x_14_*/Yuan) |
| --- | --- | --- | --- | --- | --- | --- | --- | --- | --- | --- | --- | --- | --- | --- | --- |
| Jiangan | 307.18 | 17.31 | 78.70 | 1094.49 | 1.62 | 1655.57 | 962633.3 | 11990.95 | 99.99999 | 1.314504 | 12.61517 | 14.33 | 186.3333 | 35585 | 51333.33 |
| Jianghan | 176.41 | 17.32 | 78.64 | 1094.36 | 1.63 | 1656.07 | 730066.7 | 25806.53 | 99.9 | 1.587375 | 20.07429 | 18.90 | 128 | 36230 | 51432.67 |
| Qiaokou | 128.87 | 17.33 | 78.48 | 1098.24 | 1.65 | 1657.06 | 868700 | 21684.97 | 99 | 1.513988 | 17.57727 | 21.31 | 138.6667 | 34875 | 42618.67 |
| Hanyang | 256.59 | 17.33 | 78.46 | 1097.90 | 1.65 | 1657.28 | 663666.7 | 5950.033 | 99.99 | 1.038382 | 5.67989 | 7.18 | 146 | 35478.33 | 46556.67 |
| Wuchang | 431.40 | 17.32 | 78.60 | 1096.36 | 1.63 | 1656.42 | 1282133 | 19853.41 | 100 | 1.162841 | 13.44017 | 15.09 | 174 | 55477.33 | 51131 |
| Qingshan | 282.03 | 17.32 | 78.47 | 1097.40 | 1.65 | 1658.33 | 530633.3 | 9289.799 | 96.45267 | 0.997735 | 8.623708 | 10.66 | 120.3333 | 28033.33 | 50236 |
| Hongshan | 336.69 | 17.35 | 78.17 | 1105.38 | 1.69 | 1660.22 | 1674833 | 2921.493 | 96.98 | 0.859363 | 5.638739 | 5.95 | 220.6667 | 46553 | 46741.67 |
| Dongxihu | 226.38 | 17.37 | 77.52 | 1091.79 | 1.76 | 1660.91 | 582500 | 1175.96 | 65.43667 | 0.691362 | 7.432138 | 6.86 | 184.6667 | 34057.5 | 37962.33 |
| Hannan | 452.75 | 17.60 | 76.64 | 1237.56 | 1.99 | 1646.99 | 135300 | 471.3465 | 73.45 | 0.607995 | 6.752973 | 14.43 | 74.33333 | 11958 | 31931 |
| Caidian | 80.81 | 17.43 | 76.71 | 1099.19 | 1.87 | 1661.85 | 758633.3 | 693.9756 | 39.57 | 0.553439 | 3.363501 | 3.06 | 183 | 17953.67 | 35301.67 |
| Jiangxia | 384.31 | 17.47 | 77.22 | 1172.32 | 1.84 | 1659.69 | 954233.3 | 472.7883 | 55.00667 | 0.371459 | 2.720023 | 3.49 | 302 | 42013.5 | 35100.67 |
| Huangpi | 99.45 | 17.34 | 76.41 | 1075.18 | 1.90 | 1688.13 | 1009067 | 447.1426 | 47.92333 | 0.404537 | 6.629602 | 5.85 | 321 | 63114.5 | 35287.67 |
| Xinzhou | 67.94 | 17.32 | 75.24 | 1094.79 | 2.04 | 1793.23 | 909566.7 | 621.5307 | 53.28333 | 0.391863 | 4.757516 | 4.16 | 263.6667 | 47592.67 | 32535 |
| Huangshigang | 534.32 | 17.66 | 75.64 | 1316.59 | 1.83 | 1678.40 | 233400 | 4612.648 | 69.01 | 2.026835 | 23.16284 | 29.28 | 59.66667 | 19993.33 | 40813.33 |
| Xisaishan | 252.60 | 17.71 | 75.62 | 1342.62 | 1.81 | 1669.79 | 242800 | 2148.673 | 69 | 0.59192 | 7.167039 | 8.64 | 43.66667 | 13791 | 32090.33 |
| Xialu | 619.89 | 17.68 | 75.76 | 1320.46 | 1.84 | 1670.74 | 184733.3 | 2790.534 | 68.999 | 1.421655 | 9.409112 | 8.61 | 58.66667 | 11736.5 | 38606 |
| Tieshan | 81.88 | 17.64 | 75.89 | 1297.59 | 1.86 | 1673.74 | 56900 | 1935.374 | 69.0001 | 0.670666 | 10.62916 | 10.64 | 39 | 10226 | 35096.67 |
| Yangxin | 33.69 | 18.08 | 75.58 | 1477.04 | 1.68 | 1614.37 | 911466.7 | 327.8657 | 40.51 | 0.240264 | 4.519688 | 4.77 | 488 | 99084 | 26213.33 |
| Daye | 266.04 | 17.76 | 75.76 | 1353.08 | 1.82 | 1657.90 | 841666.7 | 537.3598 | 57.48333 | 0.316361 | 3.614627 | 4.46 | 288.3333 | 67073 | 39398.33 |
| Maojian | 236.56 | 16.33 | 71.27 | 900.95 | 1.65 | 1699.39 | 425633.3 | 788.2099 | 97.75333 | 0.371922 | 13.67439 | 2.33 | 99 | 13131 | 34643.33 |
| Zhangwan | 198.52 | 16.32 | 71.34 | 896.65 | 1.66 | 1700.56 | 393633.3 | 599.1375 | 78.15667 | 0.448252 | 13.67487 | 1.11 | 112.3333 | 21009.5 | 35091.33 |
| Yunyang | 146.38 | 16.13 | 72.39 | 784.38 | 2.02 | 1732.50 | 572900 | 149.5042 | 45.59 | 0.160521 | 2.888686 | 4.40 | 235.6667 | 30049.67 | 28187 |
| Yunxi | 87.07 | 15.52 | 73.84 | 906.29 | 1.50 | 1737.78 | 433066.7 | 123.416 | 39.16667 | 0.178177 | 4.709042 | 4.87 | 159.6667 | 30581.33 | 26836.67 |
| Zhushan | 492.03 | 15.84 | 73.59 | 921.13 | 1.63 | 1623.44 | 418566.7 | 116.7224 | 40.01667 | 0.121534 | 5.249592 | 5.30 | 206 | 28326.67 | 26404.67 |
| Zhuxi | 371.71 | 15.70 | 73.44 | 936.39 | 1.65 | 1586.26 | 315233.3 | 95.2217 | 38.89333 | 0.14426 | 6.475615 | 6.02 | 216.6667 | 22323.67 | 25975 |
| Fangxian | 67.65 | 15.18 | 75.30 | 892.81 | 1.45 | 1614.55 | 401233.3 | 78.51924 | 40.56667 | 0.116402 | 6.174244 | 6.95 | 270 | 27508 | 27873.67 |
| Danjiangkou | 282.59 | 16.17 | 74.13 | 819.46 | 1.92 | 1676.26 | 447066.7 | 143.2447 | 53.73 | 0.181319 | 8.090403 | 5.78 | 152.6667 | 32359.67 | 29498.33 |
| Xiling | 823.63 | 16.76 | 76.02 | 948.52 | 1.82 | 1397.83 | 545433.3 | 6067.112 | 100 | 1.246312 | 1.052877 | 2.61 | 70 | 19605.67 | 38622 |
| Wujiagang | 1008.85 | 16.76 | 76.04 | 951.09 | 1.82 | 1399.04 | 238200 | 2609.553 | 100 | 1.251435 | 24.09634 | 17.88 | 52 | 28056.5 | 36110.67 |
| Dianjun | 467.19 | 16.76 | 76.03 | 948.43 | 1.82 | 1397.54 | 109600 | 205.6285 | 68.02333 | 0.243773 | 34.08712 | 2.54 | 20.66667 | 1686.5 | 34385 |
| Xiaoting | 358.15 | 16.82 | 76.25 | 987.10 | 1.80 | 1415.55 | 67900 | 572.8991 | 81.1 | 0.717948 | 34.08223 | 6.78 | 15 | 3486.5 | 34363.67 |
| Yiling | 244.84 | 16.77 | 76.00 | 954.33 | 1.82 | 1403.10 | 533200 | 155.926 | 55.78333 | 0.229796 | 5.172355 | 5.04 | 123 | 21896.33 | 36786.67 |
| Yuanan | 335.25 | 16.90 | 75.21 | 955.78 | 1.95 | 1520.78 | 186666.7 | 106.5449 | 51.38 | 0.154496 | 5.828742 | 7.20 | 47 | 7985.333 | 33523.33 |
| Xingshan | 186.75 | 17.29 | 70.76 | 906.55 | 1.49 | 1416.25 | 167900 | 72.15299 | 48.61 | 0.181258 | 5.462085 | 5.60 | 38 | 5972 | 29052.33 |
| Zigui | 88.69 | 16.84 | 75.34 | 1021.07 | 1.71 | 1409.08 | 359300 | 148.0429 | 41.23667 | 0.138765 | 6.594466 | 4.93 | 78.66667 | 13357 | 28080.33 |
| Changyang | 135.05 | 16.75 | 76.51 | 1033.93 | 1.71 | 1395.24 | 386066.7 | 112.885 | 36.88 | 0.216331 | 5.219931 | 4.88 | 94.33333 | 13895.33 | 28961.33 |
| Wufeng | 81.26 | 15.90 | 80.15 | 1391.40 | 1.01 | 1238.68 | 191800 | 81.72135 | 40.12667 | 0.123643 | 4.678594 | 4.76 | 50.33333 | 7100 | 26702.67 |
| Yidu | 86.21 | 16.88 | 76.75 | 1040.51 | 1.76 | 1429.64 | 388366.7 | 286.195 | 57.04 | 0.283508 | 6.433456 | 6.70 | 78.66667 | 15673 | 36971 |
| Dangyang | 48.36 | 17.02 | 75.94 | 972.33 | 1.97 | 1524.14 | 476600 | 216.7207 | 52.68 | 0.203616 | 4.598305 | 5.95 | 90 | 18016.33 | 35037 |
| Zhijiang | 49.00 | 17.17 | 77.12 | 1018.11 | 1.90 | 1529.35 | 485433.3 | 377.0859 | 57.56667 | 0.211436 | 5.775691 | 5.31 | 102.6667 | 15792.33 | 33852 |
| Xiangcheng | 44.03 | 16.63 | 72.92 | 934.34 | 3.34 | 1708.28 | 512466.7 | 797.7501 | 73.84333 | 0.330388 | 7.517442 | 8.85 | 106 | 49448 | 36567.33 |
| Fancheng | 48.61 | 16.63 | 72.92 | 933.30 | 3.32 | 1707.58 | 909933.3 | 1619.646 | 68 | 0.364752 | 4.838001 | 6.29 | 111.6667 | 53569.33 | 36437.33 |
| Xiangzhou | 42.12 | 16.63 | 72.92 | 926.12 | 3.22 | 1702.73 | 921366.7 | 373.5462 | 60.06333 | 0.235257 | 4.470524 | 4.53 | 248 | 59142.33 | 31762.33 |
| Nanzhang | 388.29 | 16.62 | 73.49 | 891.91 | 2.51 | 1656.71 | 545266.7 | 141.5215 | 44.90333 | 0.174475 | 5.030479 | 4.94 | 241.6667 | 28431.33 | 31694.33 |
| Gucheng | 166.44 | 16.17 | 74.95 | 794.29 | 1.95 | 1647.82 | 509266.7 | 200.4221 | 51.76667 | 0.153015 | 7.26581 | 7.00 | 144.3333 | 34890 | 32679 |
| Baokang | 50.19 | 16.26 | 73.62 | 889.57 | 1.85 | 1605.57 | 257666.7 | 79.9827 | 44.75 | 0.13823 | 7.039754 | 6.42 | 111.3333 | 12835.33 | 28443.33 |
| Laohekou | 54.20 | 16.12 | 75.40 | 774.54 | 1.89 | 1638.42 | 481366.7 | 457.7121 | 56.52667 | 0.247113 | 6.790369 | 5.54 | 150 | 34617.33 | 34421.33 |
| Zaoyang | 18.59 | 17.22 | 70.10 | 851.83 | 1.57 | 1572.36 | 1003667 | 306.3686 | 55.09333 | 0.181949 | 6.318447 | 4.67 | 399.6667 | 70205.33 | 35271.33 |
| Yicheng | 476.18 | 16.77 | 73.44 | 899.74 | 2.61 | 1674.73 | 526666.7 | 249.1493 | 47.45333 | 0.204205 | 4.075268 | 5.46 | 220.3333 | 30804.67 | 31896.67 |
| Liangzihu | 84.11 | 17.64 | 76.20 | 1300.67 | 1.91 | 1661.17 | 145433.3 | 301.4162 | 40.65333 | 0.088348 | 1.343901 | 2.50 | 70.66667 | 10301 | 24478.33 |
| Huarong | 110.77 | 17.42 | 76.47 | 1163.73 | 1.88 | 1697.85 | 250366.7 | 605.9213 | 52.03667 | 0.463515 | 1.339751 | 2.11 | 70.66667 | 9542.333 | 28627 |
| Echeng | 266.00 | 17.51 | 75.92 | 1226.46 | 1.89 | 1698.27 | 675633.3 | 1139.54 | 76.11667 | 0.63468 | 1.340659 | 0.64 | 236.3333 | 18927 | 33392.67 |
| Dongbao | 288.92 | 17.22 | 75.61 | 914.49 | 2.37 | 1651.95 | 372300 | 226.3222 | 71.96333 | 0.222995 | 1.045369 | 3.51 | 92.66667 | 22428.33 | 36439.33 |
| Duodao | 236.10 | 17.24 | 75.83 | 925.24 | 2.31 | 1645.16 | 328800 | 533.7662 | 73.61333 | 0.393269 | 7.353268 | 3.86 | 86 | 19202.67 | 36439.33 |
| Shayang | 43.51 | 17.47 | 76.48 | 982.50 | 2.20 | 1662.93 | 623400 | 177.1023 | 48 | 0.218699 | 3.703291 | 3.23 | 148.6667 | 18408.67 | 32751.67 |
| Jingshan | 98.36 | 17.34 | 75.35 | 969.33 | 2.18 | 1692.13 | 563866.7 | 275.8643 | 54.63333 | 0.21952 | 7.490911 | 5.13 | 148.6667 | 27040.67 | 32362.33 |
| Zhongxiang | 40.64 | 17.45 | 75.61 | 853.69 | 2.84 | 1729.45 | 1010133 | 225.0743 | 56.91 | 0.153987 | 5.153829 | 3.02 | 200.3333 | 47461 | 32872.33 |
| Xiaonan | 74.55 | 17.52 | 74.94 | 1001.14 | 1.91 | 1636.38 | 929766.7 | 921.8519 | 74.04333 | 0.459865 | 4.194833 | 1.91 | 208 | 38981.67 | 35205.33 |
| Xiaochang | 8.45 | 16.88 | 74.12 | 976.94 | 2.42 | 1734.60 | 599333.3 | 542.2174 | 42.78333 | 0.299902 | 3.640078 | 3.91 | 167.6667 | 32165 | 28647 |
| Dawu | 141.15 | 16.21 | 72.29 | 900.99 | 3.21 | 1813.17 | 624533.3 | 314.9886 | 47.35667 | 0.275653 | 5.429023 | 3.81 | 178 | 35264.33 | 28923.67 |
| Yunmeng | 18.81 | 17.36 | 75.09 | 1007.39 | 2.01 | 1665.20 | 537166.7 | 924.9394 | 53.21333 | 0.408153 | 4.511925 | 5.18 | 155.3333 | 28680 | 33415.67 |
| Yingcheng | 16.78 | 17.36 | 75.32 | 1010.15 | 2.04 | 1677.68 | 606333.3 | 577.6751 | 61.99 | 0.285427 | 5.851094 | 5.03 | 118.3333 | 24726.67 | 33961 |
| Anlu | 122.56 | 16.99 | 74.82 | 980.88 | 2.18 | 1712.28 | 583733.3 | 445.7266 | 50.13667 | 0.274641 | 4.809776 | 5.48 | 162.3333 | 28722.67 | 32443.33 |
| Hanchuan | 10.63 | 17.47 | 75.93 | 1066.74 | 1.97 | 1661.11 | 1037800 | 638.4137 | 58.01 | 0.273545 | 4.712508 | 4.23 | 393.3333 | 60594.33 | 33580.33 |
| Shashi | 328.71 | 17.47 | 78.95 | 983.60 | 1.98 | 1675.48 | 664233.3 | 1279.833 | 85.84667 | 0.409718 | 11.10359 | 10.74 | 78 | 27377.33 | 36893.33 |
| Jingzhou | 281.48 | 17.47 | 78.92 | 984.90 | 1.98 | 1673.50 | 584333.3 | 558.6361 | 75.48333 | 0.277946 | 7.10823 | 8.14 | 126 | 25125 | 36639 |
| Gongan | 58.53 | 17.52 | 78.22 | 1046.00 | 1.99 | 1605.26 | 848366.7 | 375.8824 | 52.13 | 0.244325 | 5.18299 | 5.53 | 194 | 38585.33 | 31382.67 |
| Jianli | 32.29 | 17.93 | 78.97 | 1146.21 | 2.21 | 1453.67 | 1023767 | 295.8863 | 43.25 | 0.183045 | 5.008488 | 4.62 | 335.6667 | 92339 | 29256 |
| Jiangling | 426.72 | 17.62 | 78.14 | 1078.05 | 2.04 | 1584.27 | 337166.7 | 326.7119 | 43.49 | 0.210404 | 4.108632 | 4.23 | 62.66667 | 16664.67 | 29368 |
| Shishou | 288.41 | 17.72 | 78.33 | 1120.27 | 2.07 | 1530.26 | 564733.3 | 395.7487 | 45.95 | 0.151757 | 4.361064 | 5.19 | 163.3333 | 26172 | 30745 |
| Honghu | 60.73 | 18.16 | 76.08 | 1262.73 | 1.78 | 1644.02 | 809766.7 | 321.4635 | 47.16667 | 0.12166 | 3.993516 | 4.20 | 169.6667 | 43596.33 | 30538 |
| Songzi | 235.37 | 17.27 | 77.53 | 1061.42 | 1.90 | 1533.66 | 768300 | 343.7584 | 52.63333 | 0.168018 | 4.341651 | 4.56 | 162 | 32607.33 | 31409 |
| Huangzhou | 952.64 | 17.49 | 75.91 | 1217.78 | 1.89 | 1701.77 | 396800 | 1095.013 | 76.23667 | 0.702619 | 11.07778 | 10.82 | 67.33333 | 21348.5 | 33199 |
| Tuanfeng | 312.76 | 17.40 | 75.71 | 1153.42 | 1.93 | 1736.63 | 345966.7 | 416.0003 | 39.79667 | 0.387535 | 5.287556 | 4.78 | 93 | 16675 | 26629.67 |
| Hongan | 128.85 | 17.15 | 74.48 | 1043.84 | 2.24 | 1816.95 | 609200 | 340.0673 | 43.82 | 0.199966 | 5.695995 | 5.60 | 162.3333 | 32581.33 | 27049.67 |
| Luotian | 547.42 | 17.26 | 73.43 | 1223.35 | 1.70 | 1799.96 | 552700 | 259.4909 | 43.82 | 0.220911 | 7.015258 | 4.20 | 151.6667 | 32798.33 | 26975 |
| Yingshan | 161.45 | 17.19 | 72.34 | 1271.83 | 1.48 | 1809.02 | 363566.7 | 252.6857 | 42.04667 | 0.246478 | 5.743918 | 5.52 | 147.3333 | 21941 | 26052.33 |
| Xishui | 154.90 | 17.43 | 74.74 | 1253.35 | 1.81 | 1737.75 | 878766.7 | 450.3909 | 42.15333 | 0.307127 | 5.242113 | 4.07 | 252 | 51982.67 | 28553.67 |
| Qichun | 516.10 | 17.63 | 75.19 | 1334.09 | 1.79 | 1691.09 | 781866.7 | 326.0005 | 45.43667 | 0.217471 | 5.404889 | 4.59 | 359.3333 | 65528 | 28248 |
| Huangmei | 167.45 | 17.51 | 75.36 | 1322.82 | 1.83 | 1698.98 | 869400 | 509.0641 | 46.25 | 0.259817 | 4.797945 | 4.87 | 273.3333 | 65013.67 | 29557.67 |
| Macheng | 105.95 | 17.54 | 74.62 | 1188.24 | 2.20 | 1810.20 | 880400 | 244.2828 | 47.62667 | 0.219756 | 5.081781 | 4.95 | 317 | 58961 | 30217 |
| Wuxue | 163.32 | 17.85 | 75.72 | 1405.66 | 1.78 | 1646.61 | 656000 | 528.308 | 53.08667 | 0.292261 | 5.979733 | 5.34 | 261 | 67447.33 | 31402 |
| Xianan | 743.02 | 17.80 | 76.41 | 1392.96 | 2.09 | 1626.89 | 530133.3 | 352.4823 | 69.47333 | 0.340927 | 8.472369 | 9.08 | 226.3333 | 51763.67 | 33662.67 |
| Jiayu | 39.75 | 17.88 | 76.08 | 1529.62 | 2.34 | 1607.88 | 318700 | 313.3727 | 50.33667 | 0.218075 | 4.795538 | 5.60 | 109.3333 | 16229 | 30625 |
| Tongcheng | 223.32 | 17.88 | 77.12 | 1373.41 | 1.98 | 1597.17 | 416433.3 | 364.9722 | 45.24333 | 0.211338 | 5.238211 | 5.74 | 194.3333 | 39470.67 | 28996.67 |
| Chongyang | 197.23 | 17.86 | 76.64 | 1396.56 | 2.04 | 1614.12 | 407833.3 | 207.2324 | 45.52667 | 0.185572 | 5.686114 | 5.90 | 244.6667 | 40477.33 | 27343.67 |
| Tongshan | 365.45 | 17.79 | 76.60 | 1420.91 | 1.96 | 1624.22 | 376833.3 | 140.6095 | 44.32 | 0.193553 | 4.564283 | 5.53 | 188.3333 | 45524 | 25474.67 |
| Chibi | 232.89 | 17.91 | 76.39 | 1395.56 | 2.10 | 1617.07 | 492333.3 | 285.7419 | 56.34333 | 0.26583 | 5.110439 | 6.45 | 169.3333 | 36927.33 | 31611.33 |
| Zengdu | 127.72 | 16.25 | 77.00 | 947.71 | 1.53 | 1743.40 | 638000 | 484.8024 | 70.11667 | 0.275078 | 6.900909 | 6.67 | 129.3333 | 23193.33 | 32184 |
| Suixian | 154.31 | 16.52 | 75.45 | 934.55 | 1.76 | 1718.89 | 804333.3 | 141.7827 | 43.68 | 0.148074 | 2.661286 | 2.56 | 258 | 41338.67 | 26791.67 |
| Guagshui | 119.48 | 16.53 | 73.82 | 947.96 | 2.51 | 1763.47 | 773733.3 | 292.3058 | 48.52667 | 0.202351 | 4.596137 | 4.11 | 291 | 49399 | 29130 |
| Enshi | 548.49 | 17.27 | 80.08 | 1437.02 | 0.91 | 1228.67 | 780500 | 196.7482 | 55.94 | 0.239876 | 10.08282 | 9.76 | 258.3333 | 53151 | 31624.67 |
| Lichuan | 96.16 | 13.64 | 78.80 | 1250.10 | 1.29 | 1148.93 | 671966.7 | 145.8894 | 44.20667 | 0.164273 | 7.576779 | 6.36 | 295.6667 | 69883.67 | 28986 |
| Jianshi | 244.53 | 15.86 | 77.95 | 1249.09 | 1.31 | 1188.21 | 422166.7 | 158.4115 | 41.5 | 0.177591 | 7.522913 | 5.57 | 162 | 28105 | 27347.33 |
| Badong | 156.35 | 17.64 | 71.89 | 1078.49 | 1.88 | 1427.01 | 430700 | 128.4905 | 39.03333 | 0.22109 | 5.342554 | 4.84 | 173.6667 | 27721.33 | 27773 |
| Xuanen | 290.14 | 16.42 | 79.30 | 1331.12 | 1.14 | 1210.52 | 307433.3 | 112.3249 | 37.94667 | 0.107737 | 4.793803 | 5.46 | 144.3333 | 21983 | 27117.67 |
| Xianfeng | 252.21 | 16.12 | 80.01 | 1318.27 | 1.24 | 1168.81 | 309666.7 | 122.7375 | 42.64667 | 0.132449 | 7.251118 | 6.39 | 172.6667 | 24877 | 27214.33 |
| Laifeng | 287.17 | 16.40 | 80.74 | 1384.86 | 1.18 | 1180.58 | 249166.7 | 185.6682 | 43.16667 | 0.106495 | 7.581679 | 7.20 | 105.3333 | 25518.67 | 27925.67 |
| Hefeng | 237.19 | 16.43 | 78.73 | 1270.17 | 1.26 | 1245.95 | 204833.3 | 71.42027 | 37.77667 | 0.131999 | 4.904481 | 5.07 | 97.66667 | 10328 | 27565 |
| Xiantao | 149.60 | 17.65 | 76.03 | 1118.75 | 2.05 | 1653.20 | 1140367 | 449.317 | 58.56667 | 0.256052 | 5.026842 | 6.93 | 341.3333 | 86077.33 | 31826.33 |
| Qianjiang | 113.80 | 17.58 | 76.27 | 1032.43 | 2.06 | 1648.90 | 965700 | 481.8862 | 52.23333 | 0.246072 | 4.732519 | 6.44 | 235.6667 | 51881.67 | 31828.33 |
| Tianmen | 205.08 | 17.56 | 74.72 | 951.44 | 1.98 | 1705.48 | 1267733 | 483.4986 | 54.03333 | 0.276753 | 5.140857 | 4.86 | 287 | 75193.67 | 29035.33 |
| Shennongjia | 323.06 | 16.07 | 73.76 | 919.58 | 1.60 | 1548.20 | 76533.33 | 23.527 | 48.23 | 0.094083 | 6.457705 | 6.12 | 47.66667 | 3474.333 | 28223.67 |

Table 4: Spearman correlation analysis of influenza incidence in Hubei from 2017 to 2019

| **Time** | **Temperature (*x*_1_)** | **Humidity (*x*_2_)** | **Precipitation (*x_3_*)** | **Wind speed (*x*_4_)** | **Sunshine (*x*_5_)** |
| --- | --- | --- | --- | --- | --- |
| **January** | -0.51** | 0.35** | 0.04 | -0.13* | -0.41** |
| **February** | -0.63** | 0.32** | -0.08 | -0.19** | -0.31** |
| **March** | 0.25** | -0.02 | -0.21** | -0.08 | 0.32** |
| **April** | -0.10 | 0.30** | -0.19** | -0.03 | -0.41** |
| **May** | -0.35** | -0.11 | 0.06 | -0.24** | -0.22** |
| **June** | 0.17** | -0.08 | 0.19** | -0.002 | 0.07 |
| **July** | -0.15* | 0.06 | -0.23** | -0.03 | -0.12* |
| **August** | -0.27** | 0.34** | 0.33** | -0.24** | -0.32** |
| **September** | -0.12* | 0.19** | 0.11 | -0.14* | -0.12 |
| **October** | -0.02 | 0.12* | 0.11 | -0.09 | -0.16** |
| **November** | 0.20** | -0.18** | -0.11 | -0.11 | -0.17** |
| **December** | 0.46** | 0.16** | -0.10 | -0.17** | 0.13* |
| **Monthly Total** | -0.30** | -0.11** | 0.08** | -0.24** | -0.07** |

Note: ** indicates that the significance level is 0.01, * indicates that the significance level is 0.05.


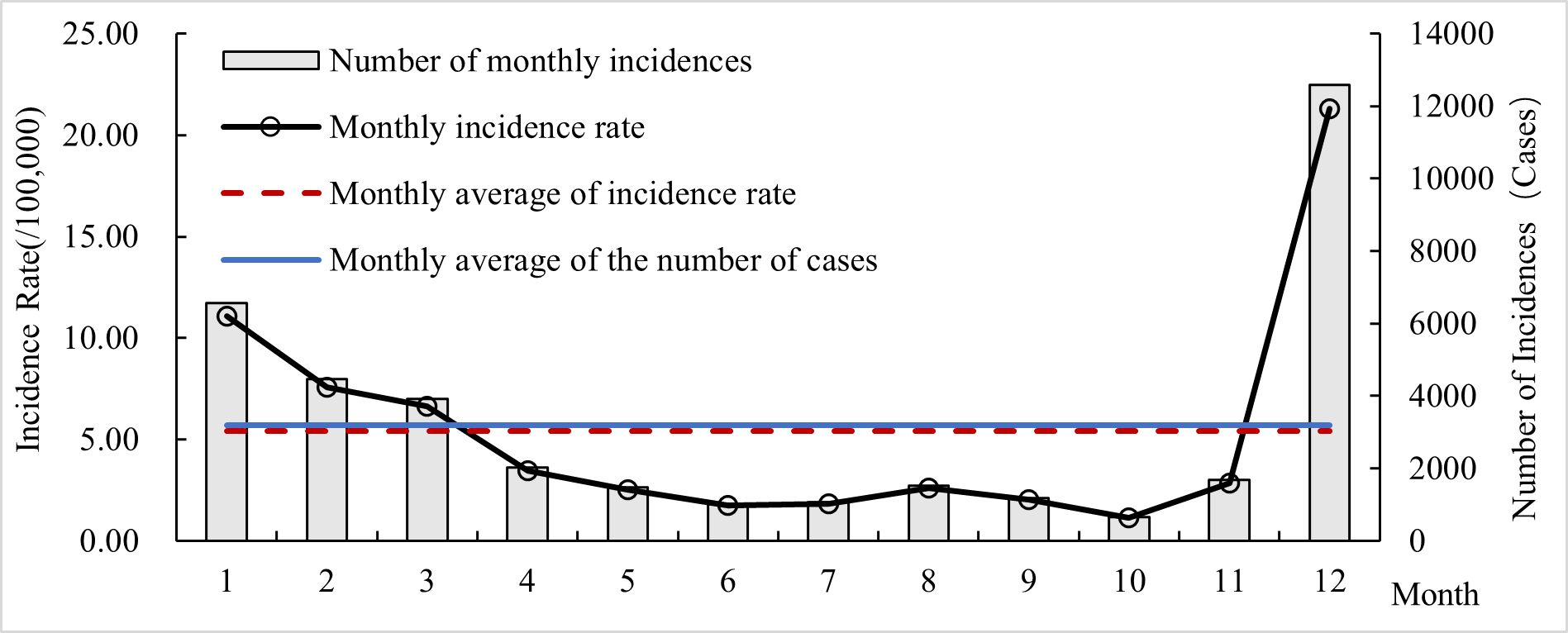


**Fig.1. Monthly changes in influenza cases and incidence rates in Hubei from 2009 to 2019**


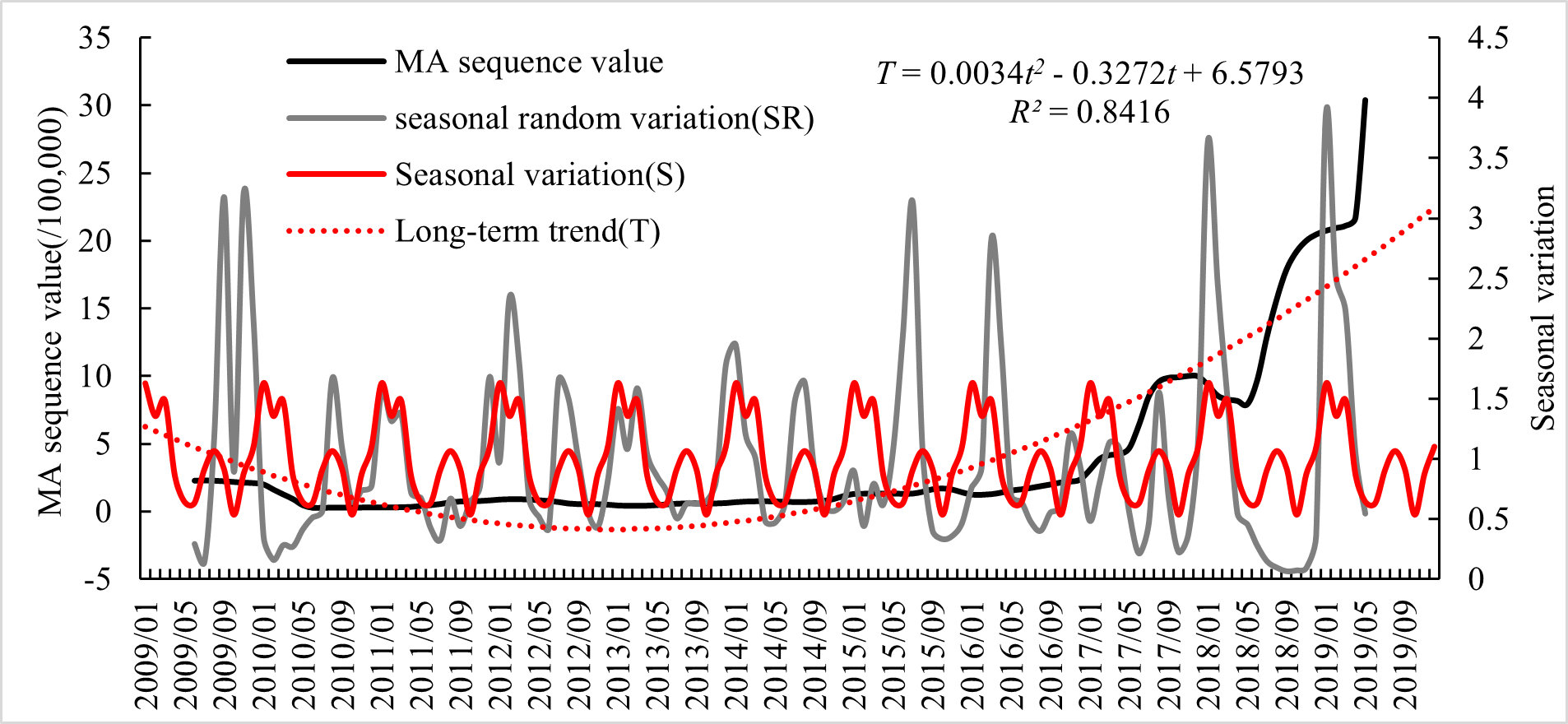


**Fig.2. Seasonal index** **and long-term trend of influenza incidence in Hubei in 2009-2019**


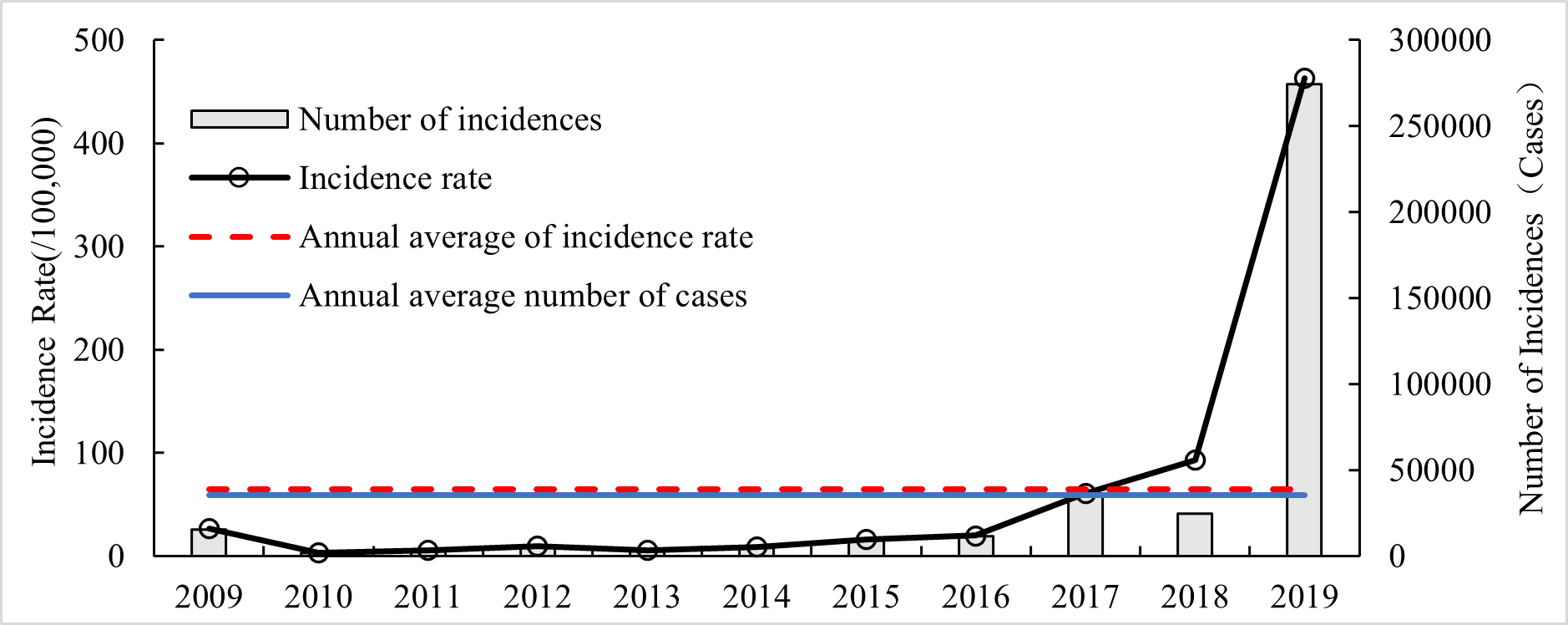


**Fig.3. Interannual changes in influenza cases and incidence rate in Hubei from 2009 to 2019**


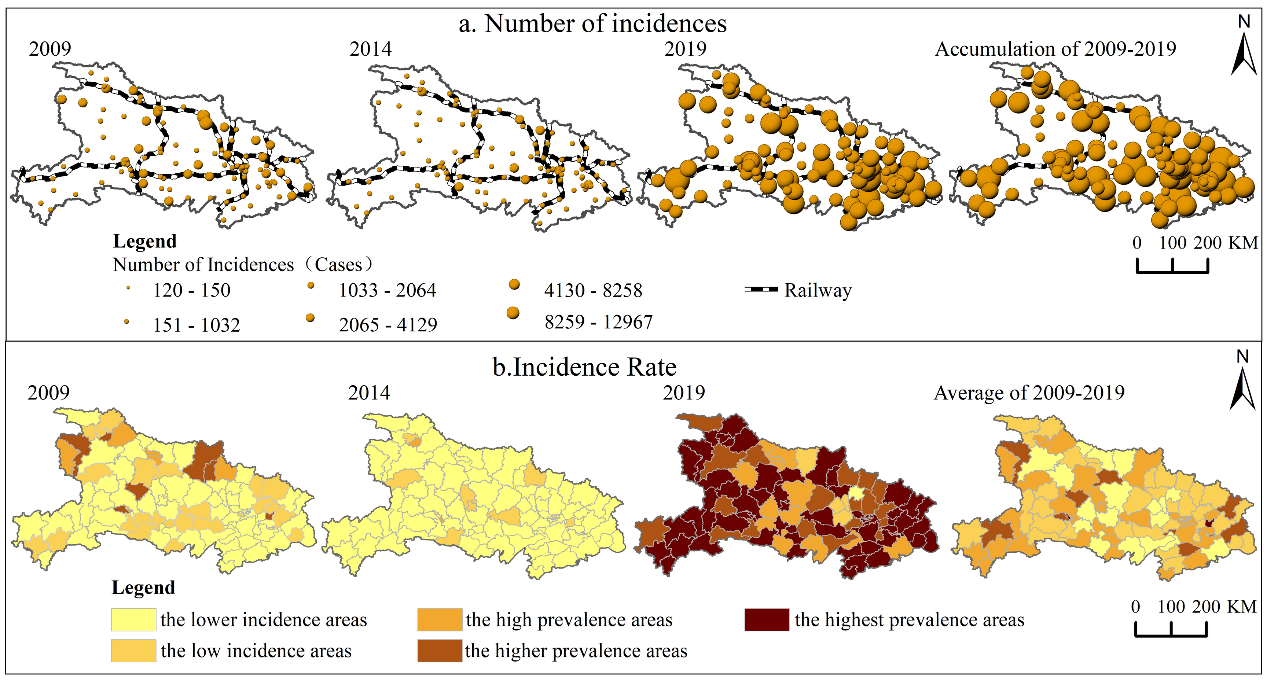


**Fig.4. Spatial distribution of Influenza Incidents in Hubei Province**

Note: The basemap came from United States Geological Survey (<https://apps.nationalmap.gov/services/>), the map boundary has not been changed. Cartographic software: ArcGIS.


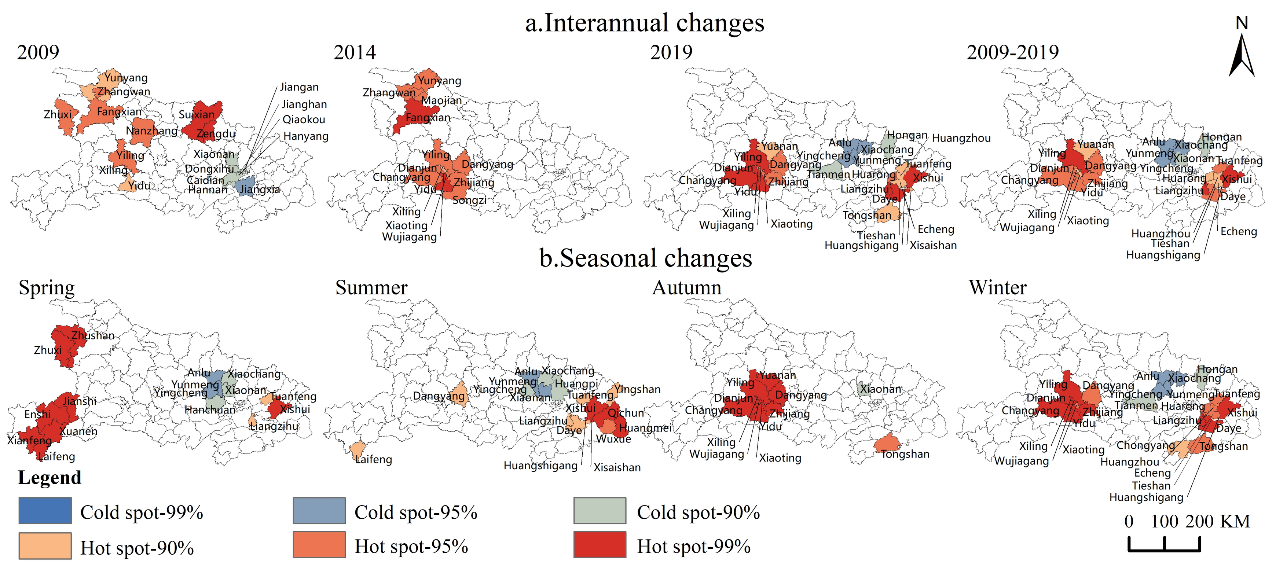


**Fig. 5.** **Interannual (a) and seasonal changes (b) of influenza hot spots in Hubei Province**

Note: The basemap came from United States Geological Survey (<https://apps.nationalmap.gov/services/>), the map boundary has not been changed. Cartographic software: ArcGIS.


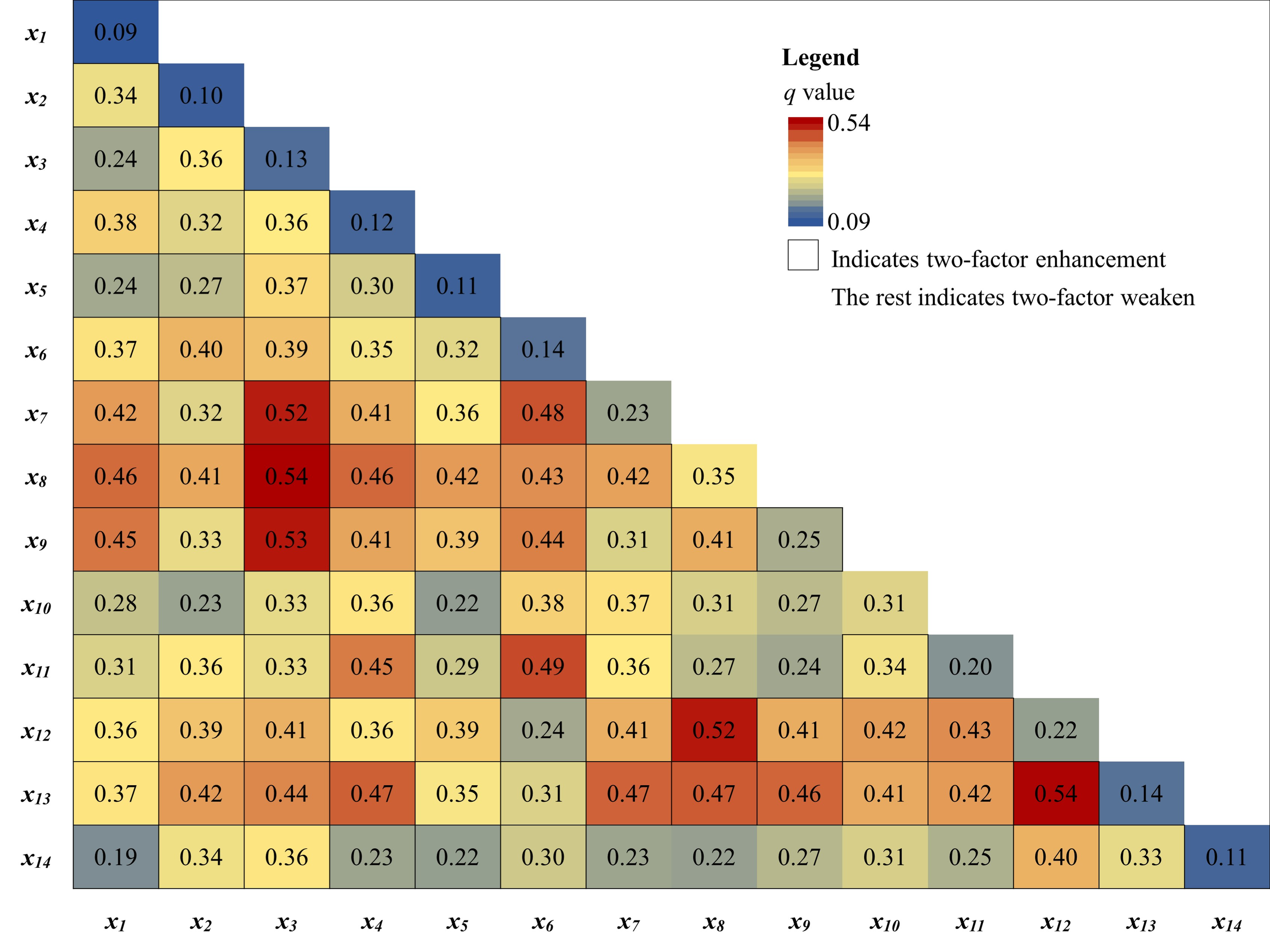


**Fig.6. Interaction hotspot maps of influencing factors on influenza incidence in Hubei Province**

Note: *x_1_*: temperature, *x_2_*: humidity, *x_3_*: precipitation, *x_4_*: wind speed, *x_5_*: sunshine, *x_6_*: permanent resident population, *x_7_*: population density, *x_8_*: urbanization rate, *x_9_*: road density, *x_10_*: number of beds per 1,000 population, *x_11_*: number of health technicians per 1,000 population, *x_12_*: number of schools, *x_13_*: number of primary school students in school, *x_14_*: per-capita disposable income of urban residents.


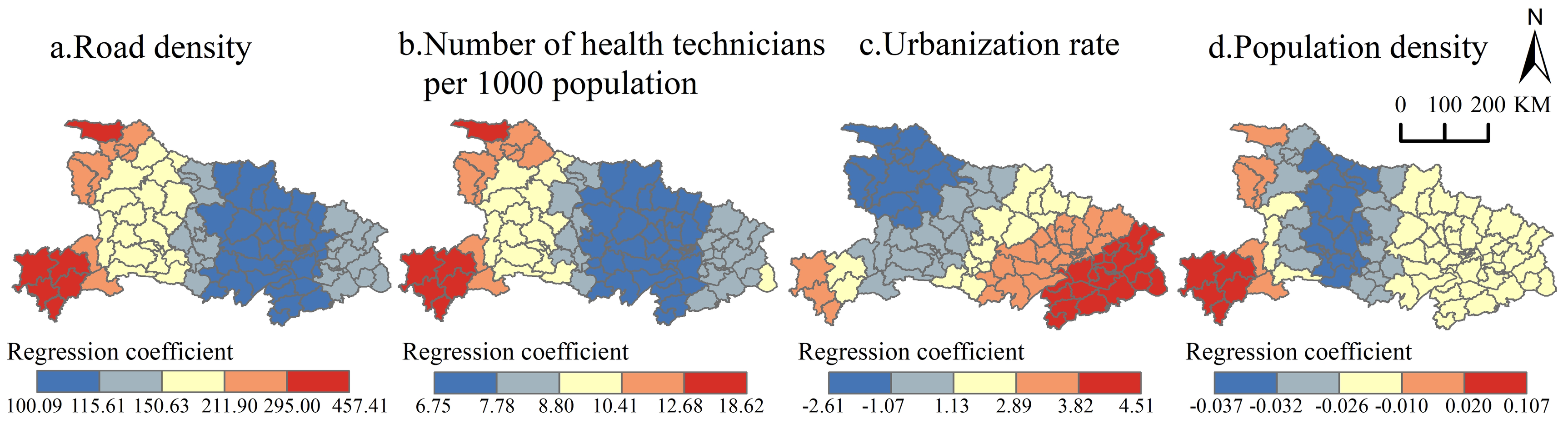


**Fig.7. Spatial variability of regression coefficients for influencing factors for influenza incidence in Hubei Province**

Note: The basemap came from United States Geological Survey (<https://apps.nationalmap.gov/services/>), the map boundary has not been changed. Cartographic software: ArcGIS.
